# The Role of Large Language Models in the Promotion of Minimally Invasive Interventional Radiologic Methods in Gynecology and Obstetrics

**DOI:** 10.3390/jcm15093234

**Published:** 2026-04-23

**Authors:** Iason Psilopatis, Julius Emons, Kleio Vrettou, Tibor A. Zwimpfer

**Affiliations:** 1 Department of Gynecology and Obstetrics, University Hospital Basel, University of Basel, 4056 Basel, Switzerland; 2 Department of Gynecology and Obstetrics, Universitätsklinikum Erlangen, Comprehensive Cancer Center Erlangen-EMN (CCC ER-EMN), Friedrich-Alexander-Universität Erlangen-Nürnberg (FAU), 91054 Erlangen, Germany; 3 Department of Cytopathology, Sismanogleio General Hospital, Sismanogleiou 1, 15126 Athens, Greece; 4 Department of Biomedicine, University Hospital and University of Basel, 4031 Basel, Switzerland

**Keywords:** large language model, uterine artery embolization, fibroid, adenomyosis, postpartum hemorrhage

## Abstract

**Background:** Minimally invasive interventional radiology (IR) offers effective, uterus-preserving treatments for several gynecologic and obstetric conditions such as uterine fibroids, adenomyosis and postpartum hemorrhage. Despite their efficacy, these methods remain underused, partly to limited awareness among clinicians and patients. Large language models (LLMs) may help bridge this gap by providing accessible, reliable information. **Objective:** To evaluate how current LLMs address knowledge gaps and promote awareness of minimally invasive IR methods in gynecology and obstetrics. **Methods:** A structured ten-question instrument was used to query three publicly available LLMs (OpenEvidence, ChatGPT, and Google Gemini). Responses were analyzed for accuracy, completeness, safety considerations, and patient-centered communication. **Results:** All three models accurately identified a range of medical, minimally invasive, and surgical treatments for uterine fibroids, adenomyosis, and postpartum hemorrhage, with OpenEvidence and ChatGPT providing more detailed and clinically nuanced responses. OpenEvidence achieved the highest scores overall, closely followed by ChatGPT, while Google Gemini scored lower, particularly in completeness and patient-centered communication. In more complex scenarios, performance differences became more pronounced, with OpenEvidence again leading, ChatGPT performing strongly, and Google Gemini lagging behind. Overall, OpenEvidence and ChatGPT demonstrated higher accuracy, completeness, and safety considerations, whereas Google Gemini showed comparatively weaker and less consistent performance. **Conclusions:** LLMs may endorse the promotion of minimally invasive IR methods in gynecology and obstetrics, but their outputs vary considerably in quality. Ongoing refinement and integration of evidence-based sources are essential before routine use in clinical practice. Therefore, effective collaboration between artificial intelligence (AI) developers and medical professionals is essential to harness this technology’s full potential.

## 1. Introduction

Gynecologic and obstetric conditions such as uterine fibroids, adenomyosis, and postpartum hemorrhage (PPH) represent a substantial global burden on women’s health [1]. Uterine fibroids, benign tumors affecting up to 70% of women by age 50, often lead to debilitating symptoms including heavy menstrual bleeding, pelvic pain, and infertility, severely impacting quality of life [2]. Adenomyosis, a condition where endometrial tissue grows into the myometrium, is similarly associated with chronic pain and abnormal bleeding, and its diagnosis and management can be complex [3]. Postpartum hemorrhage, a life-threatening obstetric emergency, remains a leading cause of maternal mortality worldwide, with timely and effective intervention being paramount for survival [4].

Historically, the management of these conditions has relied heavily on traditional, invasive surgical procedures such as hysterectomy and myomectomy [5]. While effective, these methods are associated with considerable morbidity, prolonged recovery times, and the potential loss of fertility [6]. In recent decades, minimally invasive interventional radiology (IR) has emerged as a transformative alternative, offering uterine-preserving treatments with reduced risk, faster recovery, and improved patient outcomes [7]. Procedures like uterine artery embolization (UAE) for fibroids and adenomyosis, and emergency embolization for PPH, are now established, evidence-based therapies [8,9]. However, despite their proven efficacy and benefits, the adoption and awareness of these methods remain suboptimal. A significant knowledge gap persists among both referring clinicians and patients, who often default to traditional surgical pathways due to a lack of comprehensive, accessible information [10].

The rapid and revolutionary advancement of artificial intelligence (AI), particularly in the domain of large language models (LLMs), presents an unprecedented opportunity to address this critical gap [11]. LLMs are sophisticated computational tools trained on vast datasets of text and code, enabling them to understand, process, and generate human-like language [12]. Their applications in medicine are expanding, with emerging roles in clinical decision support, medical education, and patient communication [13]. LLMs have demonstrated a remarkable capacity to synthesize complex medical literature, analyze unstructured patient data from electronic health records, and generate clear, patient-friendly information [14].

The aim of this study is to investigate whether the use of well-established LLMs could effectively promote minimal IR alternatives by proposing them—when applicable and indicated—as a standard therapeutic option for uterine fibroids, adenomyosis and postpartum hemorrhage.

## 2. Materials and Methods

### 2.1. Study Design and Setting

This cross-sectional descriptive study was designed to evaluate and compare the responses of freely accessible LLMs when prompted with common clinical questions related to the management of three gynecologic and obstetric conditions: uterine myoma, adenomyosis and postpartum hemorrhage. The study was performed in August 2025.

### 2.2. Data Sources

The investigation was conducted using three distinct LLMs: OpenEvidence, ChatGPT, and Google Gemini. These models were selected due to their widespread accessibility and different underlying software, providing a diverse sample for comparative analysis. Each model was accessed in August 2025 through its publicly available interface, without paid subscriptions (latest available free LLM version with default system settings; GPT-4o/-5, Gemini 2.5, OpenEvidence (https://www.openevidence.com/)). Each LLM was queried with the complete set of ten questions in the English language, without consistent session resetting before each query. The responses were recorded verbatim for subsequent analysis. No follow-up clarifications or iterative prompting were performed.

### 2.3. Instrument and Variables

A short questionnaire comprising ten specific clinical questions was developed for this study. Each question was carefully formulated to assess the LLMs’ ability to provide accurate, comprehensive, and up-to-date information on therapeutic options, with a particular focus on the inclusion and appropriate recommendation of minimal IR alternatives. These questions were developed based on the official recommendations of the American College of Obstetricians and Gynecologists, as well as the German Society of Gynecologists and Obstetricians, in order to ensure an assessment of the LLMs’ responses compared to the official guideline suggestions [15,16,17,18,19]. The questions were categorized into two primary areas: general treatment options and specific contraindications or considerations for IR procedures.

The first five questions focused on general therapeutic options:

- Questions 1–3: What are the therapeutic options for patients presenting with uterine fibroids and typical clinical symptoms?
- Question 4: What are the treatment options for a patient diagnosed with adenomyosis?
- Question 5: What are the treatment options for a patient with postpartum hemorrhage?

The subsequent five questions were designed to test the LLMs’ understanding of nuanced clinical scenarios and potential contraindications for minimal IR procedures:

- Question 6: What are the treatment options for a pregnant patient with uterine fibroids?
- Question 7: What are the treatment options for a patient with a uterine fibroid and a wish for future fertility?
- Question 8: What are the treatment options for a patient with a uterine fibroid and an active concomitant pelvic infection?
- Question 9: What are the treatment options for a patient with adenomyosis and a suspicious adnexal mass?
- Question 10: What are the treatment options for a patient with a pedunculated myoma?

### 2.4. Outcomes Measures

Responses were evaluated according to four predefined criteria:

1. Accuracy (concordance with established guidelines and evidence)
2. Completeness (inclusion of relevant clinical aspects)
3. Safety considerations (acknowledgement of risks and complications, emphasis on multidisciplinary care)
4. Patient-centered communication (clarity, accessibility, and balance of information)

### 2.5. Assessment and Bias Minimization

Two independent reviewers with expertise in gynecology and obstetrics assessed the responses. A 5-point Likert scale was employed for the evaluation of each response according to the four predefined criteria. A score of one point indicated poor quality, with the recommendations being inaccurate or potentially harmful. A score of two points reflected fair quality, with the information covering some helpful points but also including significant mistakes or missing details. A score of three points represented good quality, with generally accurate information that may, nevertheless, lack some important details. A score of four points indicated very good quality, where the information was accurate and clear with minor limitations. Finally, a score of five points signified excellent quality, offering complete, reliable, and fully actionable advice. The overall score was awarded based on the performance of each LLM in all four domains per question. Discrepancies were resolved by consensus discussion prior to awarding a final score to each LLM response. To reduce bias, evaluators were blinded to the model identity during the round of assessment.

### 2.6. Statistical Analysis

Descriptive statistics were used to summarize mean values across all models and cases. No inferential statistical comparisons were performed given the exploratory and qualitative nature of the study, as well as the small study sample.

## 3. Results

We evaluated 30 responses generated by the three LLMs ChatGPT, OpenEvidence and Google Gemini, to a 10-question instrument covering key aspects of minimally invasive IR procedures in gynecology and obstetrics.

### 3.1. Descriptive Findings

The evaluation of the LLM responses revealed a consistent ability across all three models to provide comprehensive overviews of treatment options for the clinical scenarios presented. However, variations in the level of detail, clinical nuance, and the prominence given to IR alternatives were observed (Supplementary Files S1–S3).

### 3.2. General Therapeutic Options (Questions 1–5)

For the initial questions on uterine fibroids, adenomyosis, and postpartum hemorrhage, all three models correctly identified and described a range of medical, non-surgical (minimally invasive), and surgical treatments (Table 1). Overall, all models successfully presented IR procedures as viable alternatives. OpenEvidence and ChatGPT, however, provided a more detailed and clinically layered response that went beyond a simple list of options. OpenEvidence received a mean score of four points for its accuracy, completeness, and safety considerations, and a mean score of three points in terms of patient-centered communication. ChatGPT received a mean score of 3.6 points for its accuracy and patient-centered communication, and a mean score of 3.8 points for its completeness and safety considerations. Google Gemini received a mean score of 3.6 points for its accuracy, a mean score of 3.4 points for its completeness and safety considerations, and a mean score of 3.2 points in terms of patient-centered communication. OpenEvidence and ChatGPT were awarded the same mean overall score (3.8 points), while Google Gemini achieved a mean overall score of 3.4 points (Table 2).

### 3.3. Contraindications for Minimal IR Procedures (Questions 6–10)

Most differences were observed in the models’ handling of complex clinical scenarios and contraindications (Table 1). For instance, OpenEvidence provided a very accurate and complete response concerning the treatment options for patients with uterine fibroids and fertility wish, whereas Google Gemini proposed UAE or radiofrequency ablation as second-line therapies, without, however, explicitly highlighting the potential reproductive risks that have been described in the literature. In general, OpenEvidence received a mean score of 4.2 points for its accuracy, a mean score of four points for its completeness, a mean score of 4.6 points for its safety considerations, and a mean score of 3.4 points in terms of patient-centered communication. ChatGPT received a mean score of four points for its accuracy and completeness, a mean score of 4.2 points for its safety considerations, and a mean score of 3.4 points in terms of patient-centered communication. Google Gemini received a mean score of 3.4 points for its accuracy, a mean score of 2.8 points for its completeness, a mean score of 3.2 points for its safety considerations, and a mean score of three points in terms of patient-centered communication. OpenEvidence was awarded a mean overall score of 4.2 points, ChatGPT a mean overall score of four points, while Google Gemini achieved a mean overall score of 3.2 points (Table 2).

## 4. Discussion

The results of this exploratory study demonstrate the varying capabilities of freely accessible LLMs in potentially providing clinical guidance related to gynecologic and obstetric conditions and promoting the role of minimally invasive IR methods in these cases. The primary finding is that while all models can accurately list minimally invasive IR therapeutic options, they differ in their ability to provide clinically responsible and nuanced guidance, particularly concerning contraindications and the appropriate clinical context for minimally invasive IR procedures. ChatGPT and OpenEvidence consistently outperformed Google Gemini in this regard, offering more explicit warnings and detailed rationales that are essential for safe clinical practice.

The clinical implications of these findings are important. The widespread use of LLMs by both patients and clinicians for quick information retrieval necessitates that these models not only provide correct information but also contextually appropriate and safe guidance. As our results show, some LLMs are more effective than others at highlighting specific situations in which IR is a preferred option, or conversely, a contraindicated one. This capability positions them as a powerful tool for promoting the appropriate utilization of IR by bridging the existing knowledge gap and facilitating shared decision-making. By making complex clinical information more accessible and understandable, LLMs can empower patients to ask informed questions and encourage referring clinicians to consider non-surgical alternatives more readily.

The performance of the LLMs in this study aligns with existing literature on the application of AI in medicine. Studies have shown that, while LLMs can serve as valuable educational tools, their output must be carefully vetted by human experts, especially when dealing with complex or high-risk clinical scenarios [20,21,22,23]. The superior performance of OpenEvidence and ChatGPT in identifying contraindications highlights the importance of the training data and architecture behind these models. Models trained on or with direct access to peer-reviewed medical literature, such as OpenEvidence, possess an inherent advantage in providing evidence-based, clinically nuanced responses [24]. The structured and explicit nature of ChatGPT’s warnings also underscores the potential for AI to be programmed for safety-first clinical communication. Importantly, OpenEvidence is a specialized medical AI platform that retrieves information from peer-reviewed literature and is marketed as a clinical decision support tool. ChatGPT and Google Gemini are general-purpose models with different training data, knowledge cutoffs, and no inherent prioritization of evidence-based sources.

A key strength of this study is its comparative design, which evaluated multiple freely accessible LLMs against a set of clinically relevant questions. This methodology allowed for a direct comparison of their strengths and weaknesses. However, this study is not without limitations. The study is based on only 30 responses. This is a very limited sample from which to draw generalized conclusions about the clinical decision-support capabilities of LLMs across the broader field of gynecology and obstetrics. In its current form, the work principally demonstrates differences in the quality of informational responses generated by three chatbot interfaces in response to the ten predefined questions. Moreover, the comparison includes different models with different training paradigms, only free versions, and no control for prompt phrasing effects. Given the variability of LLM outputs, their responses can change over time as they are updated, hence impairing the reproducibility of the results, whereas the free versions of these models may not always reflect their full capabilities. Furthermore, the study’s scope was limited to a specific set of gynecologic and obstetric conditions and did not explore the full breadth of IR applications.

Future research should focus on a longitudinal evaluation of LLM performance, under consideration of possible ethical risks (e.g., misinformation, patient misuse of LLMs), as technology evolves. It would be beneficial to expand the scope to include a wider range of clinical specialties and a larger, more diverse set of questions. Investigating the clinical impact of LLM use by both patients and clinicians, for example, by conducting a randomized controlled trial, would be a critical next step. The development of specialized, medical-focused LLMs with transparent training data and rigorous validation protocols could also address some of the current limitations.

In conclusion, our study confirms that LLMs hold potential to promote the use of minimally invasive IR by serving as a powerful tool for information dissemination and educational support. However, it also highlights the critical need for continued development and refinement to ensure that these models provide not only accurate but also clinically responsible and safe guidance. As LLMs become more integrated into the medical landscape, the collaboration between AI developers and medical professionals will be essential to harness their full potential for a paradigm shift toward less invasive, patient-centric care.

## Figures and Tables

**Table 1:** Descriptive summary of the performance of the three LLMs on general therapeutic options, as well as nuanced clinical scenarios and contraindications.

Model	Performance on General Therapeutic Options (Questions 1–5)	Performance on Nuanced Clinical Scenarios & Contraindications (Questions 6–10)
OpenEvidence	Correctly identified and described a range of treatments, providing a detailed, stepwise approach from medical therapies to surgical options. Stood out by grounding responses in cited sources from reputable journals and organizations.	Consistently provided precise and nuanced clinical details. Correctly noted that minimally invasive procedures like UAE are “less commonly used for pedunculated fibroids” due to risks like post-procedural expulsion and infection. It also correctly identified that active pelvic infection is a contraindication for elective IR procedures.
ChatGPT	Adopted a structured approach with bullet points and concise summaries. Accurately listed medical, minimally invasive (UAE and MRI-guided Focused Ultrasound), and surgical options. Noted the effectiveness of UAE for certain symptoms and highlighted its potential impact on fertility.	Demonstrated a more nuanced understanding than Google Gemini. Explicitly stated that UAE is generally avoided for pedunculated subserosal fibroids due to the risk of “stalk necrosis, detachment, and peritonitis”. Correctly identified myomectomy as the preferred surgical approach for fertility preservation.
Google Gemini	Provided a clear, well-structured response, accurately listing medical, non-surgical, and surgical treatments. Categorized non-surgical treatments like UAE, RFA, and Focused Ultrasound as “less invasive than traditional surgery”.	Gave less detailed and nuanced information, often omitting critical warnings. For example, it did not explicitly mention the potential contraindication of UAE for a pedunculated myoma, failing to highlight the risk of stalk necrosis or detachment.

**Table 2:** Evaluation of each LLM response based on accuracy, completeness, safety considerations, and patient-centered communication.

Model	Question 1	Question 2	Question 3	Question 4	Question 5
OpenEvidence	Accuracy: 5	Accuracy: 4	Accuracy: 4	Accuracy: 4	Accuracy: 3
	Completeness: 5	Completeness: 4	Completeness: 4	Completeness: 4	Completeness: 3
	Safety considerations: 4	Safety considerations: 4	Safety considerations: 4	Safety considerations: 4	Safety considerations: 4
	Patient-centered communication: 3	Patient-centered communication: 3	Patient-centered communication: 3	Patient-centered communication: 3	Patient-centered communication: 3
	Overall score: 4	Overall score: 4	Overall score: 4	Overall score: 4	Overall score: 3
ChatGPT	Accuracy: 3	Accuracy: 4	Accuracy: 4	Accuracy: 4	Accuracy: 3
	Completeness: 4	Completeness: 4	Completeness: 4	Completeness: 4	Completeness: 3
	Safety considerations: 4	Safety considerations: 4	Safety considerations: 4	Safety considerations: 4	Safety considerations: 3
	Patient-centered communication: 4	Patient-centered communication: 4	Patient-centered communication: 4	Patient-centered communication: 4	Patient-centered communication: 2
	Overall score: 4	Overall score: 4	Overall score: 4	Overall score: 4	Overall score: 3
Google Gemini	Accuracy: 4	Accuracy: 3	Accuracy: 4	Accuracy: 4	Accuracy: 3
	Completeness: 3	Completeness: 3	Completeness: 4	Completeness: 4	Completeness: 3
	Safety considerations: 3	Safety considerations: 3	Safety considerations: 4	Safety considerations: 4	Safety considerations: 3
	Patient-centered communication: 3	Patient-centered communication: 3	Patient-centered communication: 5	Patient-centered communication: 3	Patient-centered communication: 2
	Overall score: 3	Overall score: 3	Overall score: 4	Overall score: 4	Overall score: 3
**Model**	**Question 6**	**Question 7**	**Question 8**	**Question 9**	**Question 10**
OpenEvidence	Accuracy: 5	Accuracy: 5	Accuracy: 3	Accuracy: 4	Accuracy: 4
	Completeness: 5	Completeness: 5	Completeness: 3	Completeness: 3	Completeness: 4
	Safety considerations: 5	Safety considerations: 5	Safety considerations: 4	Safety considerations: 4	Safety considerations: 5
	Patient-centered communication: 4	Patient-centered communication: 3	Patient-centered communication: 3	Patient-centered communication: 3	Patient-centered communication: 4
	Overall score: 5	Overall score: 5	Overall score: 3	Overall score: 4	Overall score: 4
ChatGPT	Accuracy: 5	Accuracy: 4	Accuracy: 3	Accuracy: 4	Accuracy: 4
	Completeness: 5	Completeness: 5	Completeness: 4	Completeness: 3	Completeness: 3
	Safety considerations: 5	Safety considerations: 4	Safety considerations: 4	Safety considerations: 4	Safety considerations: 4
	Patient-centered communication: 4	Patient-centered communication: 4	Patient-centered communication: 3	Patient-centered communication: 3	Patient-centered communication: 3
	Overall score: 5	Overall score: 4	Overall score: 3	Overall score: 4	Overall score: 4
Google Gemini	Accuracy: 4	Accuracy: 3	Accuracy: 3	Accuracy: 4	Accuracy: 3
	Completeness: 4	Completeness: 2	Completeness: 2	Completeness: 3	Completeness: 3
	Safety considerations: 4	Safety considerations: 3	Safety considerations: 3	Safety considerations: 3	Safety considerations: 3
	Patient-centered communication: 3	Patient-centered communication: 3	Patient-centered communication: 3	Patient-centered communication: 3	Patient-centered communication: 3
	Overall score: 4	Overall score: 3	Overall score: 3	Overall score: 3	Overall score: 3

## Data Availability

The supporting data are presented in the Supplementary Materials.

## Supplementary Materials

The following supporting information can be downloaded at: https://www.mdpi.com/article/10.3390/jcm15093234/s1, Supplementary File S1. Google Gemini’s Responses; Supplementary File S2. ChatGPT’s Responses; Supplementary File S3. OpenEvidence’s Responses.
