# Supplementary material for: The Role of Large Language Models in the Promotion of Minimally Invasive Interventional Radiologic Methods in Gynecology and Obstetrics
Source: J Clin Med. 2026 Apr 23;15(9):3234. doi: 10.3390/jcm15093234 (PMC13163413; doi:10.3390/jcm15093234)
Supplement: Supplementary file 1 [file jcm-15-03234-s001.zip › S1-Gemini UAE.pdf]

Supplementary File 1. Google Gemini's Responses

What are the treatment options for a patient suffering from menorrhagia due to uterine fibroids?

For a patient suffering from menorrhagia (heavy menstrual bleeding) due to uterine fibroids, treatment options vary based on the patient's age, overall health, desire for future pregnancy, and the size, location, and number of fibroids. The treatment options can be broadly categorized into medical, non-surgical, and surgical.

Medical Treatments

Medical treatments are often the first line of defense, especially for managing symptoms. They do not typically shrink the fibroids but can help control the bleeding.

- **Nonsteroidal Anti-inflammatory Drugs (NSAIDs):** Medications like ibuprofen and naproxen sodium can help reduce menstrual blood loss and alleviate painful cramps.
- **Tranexamic Acid (Lysteda):** This non-hormonal medication is taken only during the days of heavy bleeding and helps reduce blood loss.
- **Hormonal Birth Control:** Oral contraceptives, a hormonal IUD (e.g., Mirena), or other hormonal methods (like the patch or ring) can regulate menstrual cycles, make periods lighter, and ease heavy bleeding.
- **Gonadotropin-Releasing Hormone (GnRH) Agonists and Antagonists:** These medications induce a temporary, menopause-like state, which can shrink fibroids and stop menstrual bleeding. However, they are generally used for a short duration (e.g., up to six months) because long-term use can lead to bone loss and other menopausal side effects.
- **Selective Progesterone Receptor Modulators (SPRMs):** These drugs, like ulipristal acetate, can reduce bleeding and shrink fibroids, and they are often used as a short-term treatment or before surgery.

Non-Surgical Procedures

These procedures are less invasive than traditional surgery and aim to reduce the fibroids or their blood supply.

- **Uterine Artery Embolization (UAE):** An interventional radiologist blocks the blood vessels that supply the fibroids with tiny particles. This "starves" the fibroids of blood, causing them to shrink over time.
- **Radiofrequency Ablation (e.g., Acessa procedure):** This minimally invasive procedure uses heat to destroy individual fibroids. It is often performed laparoscopically and is effective in shrinking fibroids and relieving symptoms.
- **Focused Ultrasound:** This non-invasive procedure uses high-intensity ultrasound waves to heat and destroy fibroid tissue. It requires no incisions.

Surgical Procedures

Surgical options are considered for more severe cases or when other treatments have failed.

- **Myomectomy:** This is the surgical removal of the fibroids while leaving the uterus intact. It is the preferred option for women who wish to preserve their fertility. The procedure can be performed in several ways:
  - **Hysteroscopic Myomectomy:** A thin instrument is inserted through the vagina and cervix to remove fibroids located inside the uterine cavity.
  - **Laparoscopic Myomectomy:** The surgeon makes a few small incisions in the abdomen to access and remove the fibroids. Robotic assistance may be used.
  - **Open (Abdominal) Myomectomy:** A larger incision is made in the abdomen to remove very large, numerous, or deeply embedded fibroids.
- **Endometrial Ablation:** This procedure involves destroying the lining of the uterus to reduce or stop menstrual flow. It is a good option for women who do not want to become pregnant in the future, as pregnancy after this procedure is not recommended and can be dangerous.
- **Hysterectomy:** This is the surgical removal of the uterus and is the only permanent cure for fibroids. It is a definitive solution for menorrhagia but means the patient can no longer have children. The ovaries may or may not be removed during the procedure.

The choice of treatment is a collaborative decision between the patient and their healthcare provider, taking into account the patient's individual circumstances and goals.

Supplementary File 1. Google Gemini's Responses

What are the treatment options for a patient with uterine fibroids and pelvic pain?

Treating uterine fibroids that cause pelvic pain requires a personalized approach, as the best option depends on the size and location of the fibroids, the severity of the pain, the patient's age, and whether they want to preserve their fertility. The treatment options can be categorized into medical, non-surgical, and surgical.

Medical Treatments

These are often the first line of defense for managing pain and other symptoms, but they typically do not shrink the fibroids themselves.

- **Pain relievers:** Over-the-counter NSAIDs like ibuprofen or naproxen can help alleviate pelvic pain and cramps.
- **Hormonal birth control:** Oral contraceptives, hormonal IUDs, patches, or rings can help regulate menstrual cycles and reduce pain associated with heavy bleeding, which often contributes to pelvic pain.
- **GnRH Agonists and Antagonists:** These medications temporarily stop the production of hormones (estrogen and progesterone), which can shrink fibroids and relieve pain. They are generally used for a limited time due to potential side effects like hot flashes, vaginal dryness, and bone loss.

Non-Surgical Procedures

These minimally invasive options aim to reduce or eliminate fibroids and their symptoms without major surgery.

- **Uterine Artery Embolization (UAE):** An interventional radiologist blocks the blood supply to the fibroids by injecting tiny particles into the arteries that feed them. This causes the fibroids to shrink and die, leading to a significant reduction in pain and other symptoms.
- **Radiofrequency Ablation (e.g., Acessa procedure):** This procedure uses heat to destroy the fibroid tissue. A small probe is inserted into the fibroid, delivering radiofrequency energy to shrink it. It is often performed laparoscopically and has a shorter recovery time compared to traditional surgery.
- **Focused Ultrasound:** This non-invasive treatment uses high-intensity ultrasound waves to heat and destroy fibroid tissue. It is guided by MRI, allowing for precise targeting of the fibroids without incisions.

Surgical Procedures

Surgery is often recommended for more severe cases or when other treatments have not been effective.

- **Myomectomy:** This is a surgical procedure to remove the fibroids while leaving the uterus intact. It is the preferred option for patients who want to preserve their ability to have children. Myomectomy can be performed in several ways:

  - **Hysteroscopic Myomectomy:** For fibroids growing into the uterine cavity, a thin instrument is inserted through the vagina and cervix to remove the fibroid.
  - **Laparoscopic Myomectomy:** This minimally invasive "keyhole" surgery involves small incisions in the abdomen to remove fibroids. It often results in less pain and a faster recovery than open surgery.
  - **Open (Abdominal) Myomectomy:** This involves a larger incision in the abdomen and is used for large, numerous, or deeply embedded fibroids. It has a longer recovery period.
- **Hysterectomy:** This is the surgical removal of the entire uterus. It is the only definitive cure for fibroids and is often considered a last resort for patients who have completed their family and want to permanently resolve their symptoms. It also means they will no longer have periods.

Supplementary File 1. Google Gemini's Responses

What are the treatment options for a patient with uterine fibroids and urinary/stool incontinence?

Uterine fibroids can cause urinary or stool incontinence by growing large enough to press on the bladder or rectum, which are located near the uterus. This pressure can lead to symptoms like frequent urination, a constant urge to urinate, difficulty emptying the bladder, constipation, or, in more severe cases, incontinence.

Treating incontinence caused by fibroids involves addressing the underlying issue—the fibroids themselves. Treatment options range from less invasive, symptom-management approaches to more definitive surgical solutions. The best course of action depends on the fibroid's size and location, the severity of the symptoms, the patient's age, and their desire for future fertility.

Non-Surgical and Minimally Invasive Options

These treatments are often a good starting point, as they aim to shrink or destroy the fibroids without major surgery.

- **Uterine Artery Embolization (UAE):** This is a popular and effective non-surgical procedure. An interventional radiologist inserts a thin catheter into an artery (usually in the leg) and guides it to the uterine arteries that supply blood to the fibroids. They then inject tiny particles to block these blood vessels. By cutting off the blood supply, the fibroids shrink and die, which can relieve the pressure on the bladder and rectum and resolve the incontinence.
- **Radiofrequency Ablation (e.g., Acessa or Sonata procedure):** This minimally invasive technique uses a small probe to deliver heat to the fibroid, destroying the tissue and causing it to shrink. It can be performed laparoscopically or through the vagina and cervix (transcervically).
- **Focused Ultrasound:** This non-invasive procedure uses high-intensity ultrasound waves to heat and destroy fibroid tissue. It is guided by an MRI, allowing for precise targeting.
- **Medications:** While not a long-term solution, certain medications can help manage symptoms. Gonadotropin-releasing hormone (GnRH) agonists and antagonists can temporarily shrink fibroids, which may offer short-term relief from pressure symptoms. However, their use is limited due to side effects.

Surgical Procedures

When non-surgical options are not sufficient or appropriate, surgery may be recommended.

- **Myomectomy:** This is a surgical procedure to remove the fibroids while preserving the uterus. It is the preferred option for patients who wish to maintain their fertility. Myomectomy can be performed in a few ways depending on the fibroids' location:
    - **Laparoscopic Myomectomy:** Uses a few small incisions in the abdomen, often with robotic assistance, to remove the fibroids.
    - **Hysteroscopic Myomectomy:** For fibroids growing inside the uterine cavity, instruments are inserted through the vagina and cervix.

  - **Open (Abdominal) Myomectomy:** Involves a larger incision in the abdomen and is used for very large, numerous, or difficult-to-reach fibroids.
- **Hysterectomy:** The surgical removal of the uterus is the only permanent cure for fibroids. It is a definitive solution for fibroid-related symptoms, including pressure on the bladder and rectum. This option is typically considered for patients who do not wish to have any more children.

It's important to note that while addressing the fibroids is the primary treatment, other strategies can help manage symptoms. These include lifestyle adjustments like avoiding bladder irritants (e.g., caffeine, alcohol), pelvic floor strengthening exercises (like Kegels), and bowel training to address constipation. A patient should consult with a healthcare professional, such as a gynecologist or an interventional radiologist, to determine the most appropriate and effective treatment plan.

Supplementary File 1. Google Gemini's Responses

What are the treatment options for a patient with adenomyosis?

Adenomyosis is a condition in which the tissue that normally lines the uterus (the endometrium) grows into the muscular wall of the uterus (the myometrium). This can cause symptoms like heavy, prolonged periods and severe pelvic pain and cramping. The treatment options for adenomyosis depend on the severity of the symptoms, the patient's age, and whether they want to preserve their fertility.

Medical Treatments

These treatments aim to manage symptoms but are generally not a permanent cure for adenomyosis.

- **Pain Medications:** Over-the-counter nonsteroidal anti-inflammatory drugs (NSAIDs) like ibuprofen or naproxen can help reduce pain and cramping associated with adenomyosis. Taking them a day or two before your period and continuing through the first few days can be especially effective.
- **Hormonal Birth Control:** Hormonal contraceptives are often a first-line treatment.
  - **Levonorgestrel-releasing Intrauterine System (IUD):** An IUD like Mirena releases a hormone that thins the uterine lining, which can significantly reduce heavy bleeding and pain.
  - **Combined Oral Contraceptives (Pill), Patch, or Ring:** These hormonal methods can help regulate cycles, reduce bleeding, and alleviate pain.
  - **Progestin-Only Pills:** These can also help manage bleeding and pain.
- **Gonadotropin-Releasing Hormone (GnRH) Agonists and Antagonists:** These medications temporarily induce a menopause-like state by stopping the production of hormones that fuel adenomyosis. This can shrink the uterus and relieve symptoms. However, they are typically used for a limited time (e.g., 3 to 6 months) due to potential side effects like hot flashes, bone loss, and vaginal dryness.
- **Tranexamic Acid:** This non-hormonal medication helps reduce heavy bleeding by promoting blood clotting. It is taken only on the days of heavy bleeding.

Non-Surgical Procedures

These minimally invasive options can be effective for some patients, particularly those who want to preserve their uterus.

- **Uterine Artery Embolization (UAE):** An interventional radiologist blocks the blood vessels that supply the uterus with tiny particles, which can reduce the blood flow to the adenomyotic tissue, causing it to shrink. This can effectively reduce both heavy bleeding and pain.
- **Endometrial Ablation:** This procedure involves destroying the uterine lining. It is a good option for women who have completed their family and suffer from heavy bleeding. However, it may not address the pain if the adenomyosis has infiltrated deep into the uterine muscle, and pregnancy after this procedure is not recommended.

Supplementary File 1. Google Gemini's Responses

Surgical Treatments

Surgery is often considered when other treatments have failed or for more severe cases.

- **Hysterectomy:** This is the definitive and only permanent cure for adenomyosis. It involves the surgical removal of the uterus. Hysterectomy is typically recommended for patients who have completed their family and want a permanent solution to their symptoms. The ovaries may or may not be removed at the same time.
- **Adenomyomectomy:** This is a more complex and less common procedure that involves the surgical removal of the adenomyotic tissue while preserving the uterus. Unlike fibroids, adenomyosis does not have a clear boundary, which makes it difficult to remove all the abnormal tissue without affecting the healthy uterine wall. This surgery is generally considered for patients with a localized form of adenomyosis (an "adenomyoma") who wish to preserve their fertility. However, the risk of recurrence and potential for complications, including uterine rupture during a future pregnancy, must be carefully considered.

Supplementary File 1. Google Gemini's Responses

What are the treatment options for a patient with postpartum hemorrhage?

Postpartum hemorrhage (PPH) is a medical emergency that requires immediate and effective treatment to prevent serious complications, including maternal mortality. The treatment approach is rapid and progresses from less to more invasive, based on the cause of the bleeding. The four main causes of PPH are often referred to as the "four Ts":

- **Tone:** The uterus fails to contract after delivery (uterine atony), which is the most common cause.
- **Trauma:** Lacerations to the cervix, vagina, or perineum.
- **Tissue:** Retained placenta or blood clots.
- **Thrombin:** A pre-existing or new-onset blood clotting disorder (coagulopathy).

Treatment is often initiated with a combination of interventions simultaneously, as time is of the essence.

Initial and Medical Treatments

The first steps in managing PPH focus on resuscitation and stopping the bleeding with medications and physical maneuvers.

- **Fundal Massage and Uterine Compression:** A healthcare provider will vigorously massage the uterus through the abdomen to encourage it to contract and expel any clots. This is often the very first intervention. Bimanual compression (one hand in the vagina pressing up on the uterus and the other on the abdomen pressing down) can also be used.
- **Uterotonic Medications:** These drugs cause the uterus to contract and are the cornerstone of PPH treatment for uterine atony.
    - **Oxytocin (Pitocin):** This is the first-line medication, given intravenously or intramuscularly.
    - **Misoprostol (Cytotec):** This can be given rectally, orally, or sublingually.
    - **Methylergonovine (Methergine):** This is given by intramuscular injection and is avoided in patients with high blood pressure.
    - **Carboprost (Hemabate):** This is given by intramuscular injection and is avoided in patients with asthma.
- **Tranexamic Acid (TXA):** Given intravenously within three hours of birth, this antifibrinolytic drug helps to stabilize blood clots and can reduce mortality from PPH.
- **Fluid Resuscitation:** The patient will be given intravenous fluids and, if necessary, blood transfusions to replace lost volume and prevent shock.

Non-Surgical Procedures

If initial measures are not successful, less invasive procedures may be used to control the bleeding.

- **Intrauterine Balloon Tamponade:** A special balloon (such as a Bakri balloon) is inserted into the uterus and inflated. The pressure from the balloon compresses the uterine walls, effectively stopping the bleeding. This is a highly effective temporary or definitive measure, particularly for uterine atony.
- **Manual Removal of the Placenta or Clots:** If the bleeding is due to retained placental tissue or large blood clots, a provider may manually remove them from the uterus.
- **Uterine Packing:** In some cases, sterile gauze or sponges are packed tightly into the uterus to create pressure and stop the bleeding.

Surgical Interventions

If the bleeding continues despite medical and non-surgical interventions, surgical options are considered.

- **Laceration Repair:** If the cause is trauma, the bleeding lacerations in the cervix or vagina are surgically sutured and repaired.
- **Uterine Compression Sutures:** Surgeons can place special sutures on the outside of the uterus (e.g., B-Lynch suture) to compress it and stop the bleeding while preserving the uterus.
- **Uterine Artery Ligation or Embolization:**
  - **Ligation:** A surgeon ties off the uterine arteries to cut off the blood supply to the uterus.
  - **Embolization:** An interventional radiologist can perform a procedure similar to UAE for fibroids, where they block the blood vessels that supply the uterus with tiny particles, starving the bleeding site of blood. This is a uterine-sparing option.
- **Hysterectomy:** In severe, life-threatening cases where all other measures have failed, a hysterectomy (surgical removal of the uterus) is the last resort. While a difficult decision, it is a definitive measure to save the mother's life.

Supplementary File 1. Google Gemini's Responses

What are the treatment options for a pregnant patient with uterine fibroids?

For a pregnant patient with uterine fibroids, treatment options are significantly limited due to the need to prioritize the health and safety of both the mother and the fetus. Most fibroids do not cause complications during pregnancy and do not require any treatment. However, if symptoms arise, the approach is typically conservative.

Conservative Management

The vast majority of fibroids during pregnancy are managed by monitoring and symptom control.

- **Monitoring:** The patient's fibroids are closely monitored with ultrasounds to track their size and location. A large fibroid may be a cause for concern if it's near the cervix or birth canal, as it could obstruct the baby's passage.
- **Pain Management:** The most common symptom of fibroids during pregnancy is pain, which can be caused by the fibroid outgrowing its blood supply (a process called "red degeneration"). Treatment for this pain is generally conservative:
  - **Bed rest and hydration:** Lying down and staying well-hydrated can help ease pain and discomfort.
  - **Pain relievers:** Over-the-counter medications like acetaminophen (Tylenol) are generally considered safe for pain relief during pregnancy. Nonsteroidal anti-inflammatory drugs (NSAIDs) like ibuprofen are typically avoided, especially in the third trimester, due to potential risks to the fetus.
  - **Other pain relief:** In severe cases, a doctor may recommend stronger pain medication or, rarely, hospital admission for pain management.

Surgical Intervention During Pregnancy (Very Rare)

Surgical removal of fibroids (myomectomy) is rarely performed during pregnancy due to the high risk of hemorrhage, uterine rupture, and miscarriage. It is generally considered only in extreme and rare circumstances, such as:

- **Intractable pain:** When a patient is in severe, debilitating pain from a degenerating fibroid that does not respond to other pain management strategies.
- **Rapid growth:** If a fibroid is growing extremely fast and posing a significant risk to the pregnancy.
- **Lower uterine segment fibroids:** A fibroid in the lower part of the uterus that is blocking the birth canal and making vaginal delivery impossible. In this situation, a myomectomy may be considered at the time of a Cesarean section, though this is also associated with a high risk of significant bleeding and is generally avoided.

Management During Delivery

The presence of fibroids can influence the delivery plan.

- **Cesarean Section:** A C-section may be recommended if a large fibroid is blocking the birth canal or if it's causing the baby to be in an abnormal position (e.g., breech).
- **Postpartum Care:** After delivery, most fibroids tend to shrink on their own as hormone levels return to normal. If the fibroids continue to cause symptoms after pregnancy, definitive treatment options can be discussed at that time, such as myomectomy or other procedures.

It's important to understand that most fibroids do not cause complications during pregnancy and the primary approach is watchful waiting. The specific management plan will be determined by a healthcare provider, such as a high-risk pregnancy specialist (maternal-fetal medicine specialist), based on the individual patient's circumstances.

Supplementary File 1. Google Gemini's Responses

What are the treatment options for a patient with uterine fibroids and fertility wish?

For a patient with uterine fibroids who desires to preserve their fertility, the treatment approach is carefully chosen to manage symptoms and improve the chances of a successful pregnancy while avoiding procedures that would damage or remove the uterus. The decision depends heavily on the fibroid's size, number, and location, as some fibroids (especially those that distort the uterine cavity) are more likely to interfere with fertility.

Myomectomy: The Gold Standard

Myomectomy is the primary surgical treatment for uterine fibroids in patients who want to become pregnant. This procedure surgically removes the fibroids while leaving the uterus intact. Myomectomy can significantly improve a patient's chances of conceiving and having a successful pregnancy, especially when the fibroids are causing infertility or recurrent miscarriage by distorting the uterine cavity.

There are different types of myomectomy, chosen based on the fibroids' location:

- **Hysteroscopic Myomectomy:** This is a minimally invasive procedure for fibroids that grow inside the uterine cavity (submucosal fibroids). A thin instrument is inserted through the vagina and cervix to shave or remove the fibroids. No external incisions are required, and recovery is typically quick.
- **Laparoscopic/Robotic Myomectomy:** For fibroids growing in the uterine wall (intramural) or on the outside of the uterus (subserosal), this approach uses a few small incisions in the abdomen. It is less invasive than open surgery and leads to a faster recovery with less pain.
- **Open (Abdominal) Myomectomy:** This involves a larger incision in the abdomen and is used for very large, numerous, or deeply embedded fibroids that are not suitable for a laparoscopic approach. While more invasive, it allows the surgeon to more easily access and remove complex fibroids.

It's important to note that after a myomectomy, the uterine wall needs time to heal. Patients are often advised to wait for a certain period (e.g., three to six months) before attempting to conceive.

Other Treatment Options

While myomectomy is the main fertility-sparing surgery, other options may be considered, but with caution regarding fertility outcomes.

- **Gonadotropin-Releasing Hormone (GnRH) Agonists:** These medications can temporarily shrink fibroids, which can make a myomectomy easier and less bloody. However, they are not a long-term solution as fibroids can regrow after the medication is stopped, and their use is not a standalone treatment for fertility preservation.
- **Uterine Artery Embolization (UAE):** This procedure involves blocking the blood supply to the fibroids, causing them to shrink. While effective for symptom management, the impact of UAE on future fertility is not fully understood. Some studies have shown successful pregnancies after the

procedure, but others suggest it may negatively affect ovarian function and is therefore generally not recommended as a first-line treatment for patients actively seeking to conceive.

- **Radiofrequency Ablation (RFA):** This procedure uses heat to destroy fibroid tissue. It is a minimally invasive option. While it can preserve the uterus, more research is needed to fully understand its effects on fertility and the ability to safely carry a pregnancy.
- **Fertility Preservation:** In some cases, a doctor may discuss fertility preservation options, such as egg or embryo freezing, before a definitive fibroid treatment. This can provide a backup plan for patients concerned about the potential impact of their fibroids or treatment on their reproductive future.

When Is Treatment Not Necessary?

Sometimes, no treatment is the best option. If fibroids are not causing symptoms like heavy bleeding, pain, or infertility, a "watchful waiting" approach may be taken, with regular monitoring to ensure they don't grow or cause issues. This is especially true for fibroids that are small and located on the outside of the uterus (subserosal), as they are less likely to interfere with pregnancy.

The choice of treatment is a critical discussion between the patient and their healthcare team, which may include a gynecologist, a reproductive endocrinologist, and a maternal-fetal medicine specialist, to create a plan that aligns with the patient's fertility goals.

Supplementary File 1. Google Gemini's Responses

What are the treatment options for a patient with uterine fibroids and active pelvic infection?

An active pelvic infection, such as pelvic inflammatory disease (PID), is a significant complication for a patient with uterine fibroids. This co-occurrence changes the standard treatment plan, as any invasive procedure carries a high risk of spreading the infection, potentially leading to a more severe and life-threatening condition like sepsis.

Therefore, the immediate and primary focus is to treat and resolve the infection before considering any definitive treatment for the fibroids.

1. Initial Treatment: Addressing the Infection

- **Antibiotics:** The first and most critical step is to administer a course of antibiotics to clear the infection. The specific type and route of administration (oral or intravenous) depend on the severity of the infection. For severe cases, hospitalization and IV antibiotics are necessary.
- **Rest and Symptom Management:** The patient will be advised to rest and may be given pain relievers to manage symptoms related to both the infection and the fibroids.

No surgical or minimally invasive procedures for the fibroids should be performed while the infection is active. Doing so could lead to a massive pelvic abscess, sepsis, or even death.

2. Treatment for Fibroids After Infection is Cleared

Once the infection has been successfully treated and a healthcare provider confirms that there is no remaining active infection, the focus can shift back to the uterine fibroids. The choice of treatment for the fibroids will then follow the standard guidelines, which depend on the patient's symptoms, age, and fertility wishes.

Possible options include:

- **Medical Management:** If fibroid symptoms are mild or if the patient is nearing menopause, a "watchful waiting" approach might be taken. Medications can be used to manage symptoms like heavy bleeding (e.g., hormonal birth control, tranexamic acid) or to temporarily shrink the fibroids (e.g., GnRH agonists).
- **Surgical Options (Once Infection is Resolved):**
  - **Myomectomy:** If the patient wishes to preserve their fertility, a myomectomy (surgical removal of the fibroids) would be the recommended option. This can be performed via laparoscopy, hysteroscopy, or open abdominal surgery, depending on the fibroid's characteristics.
  - **Hysterectomy:** For patients who have completed their family and want a definitive cure for both their fibroid symptoms and a potential recurrence of infection, a hysterectomy (removal of the uterus) is an option.
- **Minimally Invasive Procedures (Once Infection is Resolved):**

  - **Uterine Artery Embolization (UAE):** This procedure involves blocking the blood supply to the fibroids, causing them to shrink. However, some sources advise against UAE in patients with a history of recent or chronic pelvic infection due to a theoretical risk of reactivating the infection.
  - **Radiofrequency Ablation (RFA):** This treatment uses heat to destroy fibroid tissue and may be an option, though, like UAE, the provider will carefully assess the risk of post-procedural infection.

In summary, the presence of an active pelvic infection completely changes the treatment priority. The infection must be treated first and foremost. Only after a full and successful course of antibiotics and a clinical assessment can a doctor and the patient safely consider treatments for the uterine fibroids themselves.

Supplementary File 1. Google Gemini's Responses

What are the treatment options for a patient with adenomyosis and a suspicious adnexal mass?

When a patient is diagnosed with both adenomyosis and a suspicious adnexal mass (a growth on the ovary or fallopian tube), the treatment approach becomes more complex. It's crucial to address both conditions, but the priority is to first determine the nature of the adnexal mass, as it could be benign or malignant. The presence of a suspicious mass significantly influences the entire treatment plan.

1. Diagnosis and Evaluation of the Adnexal Mass

The first and most important step is to thoroughly evaluate the adnexal mass. This typically involves:

- **Advanced Imaging:** A high-resolution transvaginal ultrasound is the primary tool. It can help characterize the mass (e.g., solid, cystic, mixed) and look for features that might suggest malignancy (e.g., irregular borders, solid components, blood flow within the mass).
- **Magnetic Resonance Imaging (MRI):** An MRI can provide more detailed information about the mass and the uterus, helping to confirm the diagnosis of adenomyosis and better characterize the adnexal mass.
- **Tumor Markers:** Blood tests, particularly for CA-125, may be ordered. Elevated levels can sometimes be a sign of ovarian cancer, but they can also be high in benign conditions like adenomyosis and endometriosis.
- **Consultation with Specialists:** The patient will likely be referred to a gynecologic oncologist, a surgeon who specializes in female reproductive cancers. This is a critical step to ensure a proper and safe management plan is created.

2. Treatment Options Based on the Diagnosis

The treatment plan for both adenomyosis and the adnexal mass will be determined by the findings from the initial evaluation.

If the Adnexal Mass is Highly Suspect for Malignancy

- **Surgical Excision:** The primary treatment is surgical removal of the mass. This is often done via laparoscopy or an open procedure, depending on the mass's size and suspicion level.
- **Hysterectomy:** A hysterectomy (removal of the uterus) will almost certainly be performed at the same time to definitively treat the adenomyosis and prevent future issues.
- **Staging and Further Treatment:** If the mass is confirmed to be malignant, the surgeon will also perform "staging" (checking for cancer spread), which may include removing the other ovary, the fallopian tubes, lymph nodes, and a sample of other abdominal tissues. The patient may then require chemotherapy or other cancer treatments.

In this scenario, the definitive treatment for adenomyosis is a secondary but important benefit of the necessary cancer surgery.

Supplementary File 1. Google Gemini's Responses

If the Adnexal Mass is Determined to be Benign (e.g., an Ovarian Cyst or Endometrioma)

If the mass is confirmed to be benign, the treatment plan can be tailored to address both conditions while preserving fertility if desired.

- **Surgical Excision:** A laparoscopy may be performed to remove the adnexal mass while leaving the ovary intact (if possible). At the same time, the surgeon can confirm the diagnosis of adenomyosis.
- **Combined Treatment:** After the surgery, or if surgery is not immediately necessary, the adenomyosis can be managed with hormonal treatments.
    - **Hormonal IUD:** A levonorgestrel-releasing IUD can be very effective in managing the heavy bleeding and pain associated with adenomyosis.
    - **Gonadotropin-Releasing Hormone (GnRH) Agonists:** These medications can temporarily relieve symptoms by inducing a menopause-like state.
    - **Other Hormonal Medications:** Combined oral contraceptives or other hormonal therapies can also be used to manage symptoms.
- **Hysterectomy:** If the patient has completed their family and symptoms from both the adnexal mass (if it returns) and the adenomyosis are severe, a hysterectomy would be the definitive cure. This is often the preferred option for patients with severe symptoms who no longer desire fertility.
- **Watchful Waiting:** For a patient with a small, benign adnexal mass and mild adenomyosis symptoms, the doctor may recommend monitoring both conditions over time, as some cysts resolve on their own and adenomyosis symptoms often improve after menopause.

The co-occurrence of these two conditions requires a careful, methodical approach that prioritizes ruling out malignancy. The patient's age, symptoms, and desire for future fertility are all crucial factors in determining the final, personalized treatment plan.

Supplementary File 1. Google Gemini's Responses

What are the treatment options for a patient with pedunculated uterine fibroids?

Pedunculated uterine fibroids are a specific type of fibroid that grows on a stalk or stem, which can be attached either to the outside of the uterus (subserosal) or the inside of the uterine cavity (submucosal). The treatment options for these fibroids depend on their location, size, and whether they are causing symptoms. In many cases, pedunculated fibroids are asymptomatic and may not require any treatment.

Key Considerations for Treatment

- **Symptoms:** Treatment is typically recommended if the fibroid is causing symptoms such as pelvic pain, pressure, or heavy bleeding.
- **Torsion:** A unique and serious risk with pedunculated fibroids is that the stalk can twist (torsion), cutting off the blood supply. This is a medical emergency that causes severe, acute pain and requires immediate surgical intervention.
- **Fertility:** The patient's desire for future pregnancy is a crucial factor, as some treatments preserve fertility while others do not.

Treatment Options

1. Surgical Procedures

Surgery is often the most direct and effective way to treat pedunculated fibroids, especially if they are symptomatic or causing complications like torsion.

- **Myomectomy:** This is the preferred treatment for patients who wish to preserve their uterus and fertility. It involves surgically removing the fibroid while leaving the rest of the uterus intact.
    - **Laparoscopic/Robotic Myomectomy:** For pedunculated fibroids on the outside of the uterus (subserosal), this minimally invasive approach is ideal. It involves a few small incisions in the abdomen, which allows the surgeon to use a laparoscope to access and remove the fibroid. This procedure typically has a faster recovery time than open surgery.
    - **Hysteroscopic Myomectomy:** For pedunculated fibroids that are in the uterine cavity (submucosal), this is the standard procedure. A thin instrument is inserted through the vagina and cervix to remove the fibroid without any external incisions.
- **Hysterectomy:** This is the surgical removal of the entire uterus. It is a definitive cure for fibroids and is an option for patients who have completed their family and want a permanent solution to their symptoms.

2. Non-Surgical and Minimally Invasive Procedures

These options are generally less common for pedunculated fibroids, but may be considered in certain cases.

- **Uterine Artery Embolization (UAE):** This procedure blocks the blood vessels that supply the fibroids. While effective for other types of fibroids, UAE may be less successful for pedunculated fibroids because their primary blood supply comes from the stalk, which can be difficult to target. Additionally, there is a

small risk that the fibroid could detach from the uterus and become a "parasitic" fibroid, which may require further surgery.
- **Radiofrequency Ablation (RFA):** This treatment uses heat to destroy fibroid tissue. It is generally not the first-line treatment for pedunculated fibroids due to the risk of damaging surrounding organs, as the fibroid is on a stalk.

3. Medical Management

Medications are typically used to manage symptoms, not to shrink the fibroid itself. They are a good option for patients with mild symptoms or those who are not candidates for surgery.

- **Pain Relievers:** Over-the-counter NSAIDs like ibuprofen can help manage pelvic pain and cramping.
- **Hormonal Birth Control:** Birth control pills or hormonal IUDs can help control heavy bleeding, a common symptom of fibroids.
- **GnRH Agonists:** These medications can temporarily shrink fibroids, which may make surgery easier. However, they are not a long-term solution.

Watchful Waiting

For many patients with asymptomatic pedunculated fibroids, "watchful waiting" is the best approach. The fibroids are monitored with regular ultrasounds, and treatment is only initiated if symptoms develop or if there is a concern about potential complications like torsion.
