# Supplementary material for: The Role of Large Language Models in the Promotion of Minimally Invasive Interventional Radiologic Methods in Gynecology and Obstetrics
Source: J Clin Med. 2026 Apr 23;15(9):3234. doi: 10.3390/jcm15093234 (PMC13163413; doi:10.3390/jcm15093234)
Supplement: Supplementary file 1 [file jcm-15-03234-s001.zip › S2-Chat UAE.pdf]

Supplementary File 2. ChatGPT's Responses

What are the treatment options for a patient suffering from menorrhagia due to uterine fibroids?

Menorrhagia (heavy menstrual bleeding) due to **uterine fibroids (leiomyomas)** can be managed with **medical, minimally invasive, or surgical approaches**, depending on severity, symptoms, fertility wishes, and fibroid characteristics (size, number, location).

Here's a structured overview:

---

1. Medical Management (first-line if symptoms are moderate, patient desires fertility, or surgery is contraindicated)

- **Hormonal therapies:**
  - **Levonorgestrel-releasing intrauterine device (LNG-IUD, e.g., Mirena®)** → Reduces bleeding, improves anemia, may shrink fibroids slightly.
  - **Combined oral contraceptives (COCPs)** → Cycle regulation, reduction in blood loss.
  - **Oral/injectable progestins** (medroxyprogesterone acetate, norethindrone, depot injections) → Decrease bleeding, but side effects can limit long-term use.
  - **Gonadotropin-releasing hormone (GnRH) agonists/antagonists** (e.g., leuprolide, relugolix) → Induce hypoestrogenism, shrink fibroids, reduce bleeding. Usually short-term use (e.g., preoperative or bridging to menopause) due to bone loss and menopausal symptoms.
  - **Selective progesterone receptor modulators (SPRMs, e.g., ulipristal acetate*)** → Control bleeding, reduce fibroid size. (*Note: restricted/withdrawn in some regions due to rare hepatotoxicity—local guidelines apply.)
- **Non-hormonal therapies:**
  - **Tranexamic acid** → Antifibrinolytic, taken during menses, reduces blood loss.
  - **NSAIDs (e.g., mefenamic acid)** → Reduce prostaglandins, help with pain and moderate bleeding.

---

2. Minimally Invasive Procedures

- **Uterine artery embolization (UAE):**
  - Embolization of uterine arteries → fibroid ischemia → shrinkage.
  - Effective for bleeding and bulk symptoms.
  - Preserves uterus but may affect fertility and pregnancy outcomes.
- **MRI-guided focused ultrasound surgery (MRgFUS):**
  - Noninvasive thermal ablation of fibroids.
  - Not widely available; good for selected patients.

---

3. Surgical Options

- **Myomectomy** (removal of fibroids, uterus preserved):
  - Hysteroscopic (for submucosal fibroids), laparoscopic, or open approach depending on size and number.
  - Preferred in women desiring future fertility.
  - Recurrence possible.
- **Hysterectomy** (definitive treatment):
  - For women who have completed childbearing and want a permanent solution.
  - Eliminates recurrence risk and resolves menorrhagia.

✅ **Choice of treatment depends on:**

- Severity of bleeding and anemia
- Age and proximity to menopause
- Desire for fertility or uterine preservation
- Size, number, and location of fibroids
- Patient preference and comorbidities

Would you like me to make you a **treatment algorithm (stepwise flowchart)** for fibroid-related menorrhagia that you could use as a quick reference in clinical practice?

Supplementary File 2. ChatGPT's Responses

What are the treatment options for a patient with uterine fibroids and pelvic pain?

Great question—pelvic pain with **uterine fibroids** is usually due to mass effect (pressure on adjacent organs), degeneration, or associated dysmenorrhea. The treatment options overlap with those for menorrhagia but the emphasis shifts depending on **pain vs bleeding** and **fertility wishes**.

---

1. Medical Management

- **NSAIDs (e.g., ibuprofen, mefenamic acid)** → First-line for pelvic pain/dysmenorrhea, especially if mild/moderate.
- **Hormonal therapies** (aimed at reducing fibroid activity, uterine volume, and associated pain):
    - **Combined oral contraceptives (COCPs)** → Reduce dysmenorrhea and control cycles.
    - **Progestins** (oral, injectable, LNG-IUS) → May help pain, particularly if linked to heavy bleeding.
    - **GnRH agonists/antagonists** (e.g., leuprolide, relugolix) → Shrink fibroids, improve pressure symptoms and pain, used short-term (pre-surgery or bridging to menopause).
    - **Selective progesterone receptor modulators (SPRMs, e.g., ulipristal acetate*)** → Reduce fibroid size and pain, but usage is restricted in some countries due to hepatic safety concerns.

---

2. Minimally Invasive Options

- **Uterine Artery Embolization (UAE):**
    - Effective for both bleeding and bulk-related pelvic pain/pressure.
    - May cause post-embolization pain initially.
    - Uterus preserved, but potential impact on fertility.
- **MRI-guided Focused Ultrasound (MRgFUS):**
    - Noninvasive thermal ablation, effective for pain and pressure symptoms.
    - Limited availability and patient selection criteria apply.

---

3. Surgical Options

- **Myomectomy** (uterus-preserving):
    - Removes fibroids, relieves pain from bulk/pressure or degeneration.
    - Approaches: hysteroscopic (submucosal), laparoscopic, or open.
    - Suitable for women desiring fertility.
    - Risk of recurrence.

- **Hysterectomy**:
  - Definitive treatment for fibroid-related pain.
  - Recommended for women not desiring future fertility and with severe or refractory symptoms.

✅ **Summary of approach for fibroid-related pelvic pain:**

- **Mild pain, fertility desired** → NSAIDs ± hormonal therapy, myomectomy if refractory.
- **Severe pain, fertility desired** → Myomectomy (laparoscopic/open depending on size/location).
- **No fertility desire** → Hysterectomy or UAE (depending on patient preference for uterine preservation).
- **Poor surgical candidate** → UAE or medical therapy.

Would you like me to prepare a **comparison table (treatment option vs effect on pain, fertility, bleeding, recurrence, invasiveness)**? That could be handy for quick patient counseling.

Supplementary File 2. ChatGPT's Responses

What are the treatment options for a patient with uterine fibroids and urinary/stool incontinence?

That's a very important nuance — **urinary or stool incontinence from fibroids** is less common than bleeding/pain, but when present it usually means the fibroids are causing **mass effect (compression of bladder, urethra, rectum, or pelvic floor nerves)**.

The treatment strategy here shifts more toward **definitive reduction or removal of bulk** rather than just controlling bleeding.

---

1. Medical Management (limited role)

- **GnRH agonists/antagonists** (e.g., leuprolide, relugolix):
→ Temporarily shrink fibroids, may relieve pressure-related incontinence.
→ Typically used **short-term** (preoperative or bridging to menopause).
- **Hormonal therapies (OCPs, progestins, LNG-IUS)**:
→ Good for bleeding, but **do not significantly reduce bulk**, so not very effective for compression symptoms.
- **NSAIDs / Tranexamic acid**:
→ Symptomatic for pain/bleeding only, no effect on pressure.

📌 Bottom line: **Medical therapy is usually inadequate** if incontinence is due to bulk effect.

---

2. Minimally Invasive Procedures

- **Uterine Artery Embolization (UAE):**
→ Can shrink fibroids and relieve bulk symptoms, including urinary frequency, urgency, and sometimes incontinence.
→ Fertility impact is uncertain — caution in women desiring pregnancy.
- **MRI-guided Focused Ultrasound Surgery (MRgFUS):**
→ Noninvasive ablation, may reduce pressure symptoms if fibroids shrink sufficiently.
→ Limited availability.

---

3. Surgical Options (most effective for pressure symptoms like incontinence)

- **Myomectomy** (uterus preserved):
  - Removes fibroids, relieves compression.
  - Best for women desiring fertility.

    - Approach depends on fibroid location/size (laparoscopic, open).
- **Hysterectomy** (definitive):
    - Eliminates fibroids and mass effect.
    - Best option for severe or refractory cases where fertility is **not** a priority.
    - Provides permanent resolution.

✅ **Summary by patient profile:**

- **Fertility desired:** Myomectomy ± short course of GnRH analogues for shrinkage before surgery.
- **No fertility desire, severe bulk/incontinence symptoms:** Hysterectomy (definitive).
- **Not surgical candidate:** UAE (second-line) or temporary GnRH therapy.

Would you like me to also outline **how to distinguish true fibroid-related incontinence from coexisting stress/urge incontinence** (since sometimes fibroids are coincidental, and the incontinence is urogynecological in origin)?

Supplementary File 2. ChatGPT's Responses

What are the treatment options for a patient with adenomyosis?

Great question — **adenomyosis** (endometrial glands and stroma within the myometrium) often presents with **heavy menstrual bleeding, dysmenorrhea, chronic pelvic pain, and sometimes infertility**.

Unlike fibroids, adenomyosis tends to be **diffuse** and harder to surgically remove without hysterectomy, so management focuses on **symptom control** and **uterus preservation vs definitive cure** depending on the patient's age and fertility wishes.

---

1. Medical Management (first-line if fertility desired or surgery is not an option)

- **NSAIDs**
  - For dysmenorrhea and pelvic pain, especially in mild disease.
- **Hormonal therapies:**
  - **Levonorgestrel-releasing IUD (LNG-IUS, e.g., Mirena®)** → First-line; reduces menorrhagia and dysmenorrhea, effective for many patients.
  - **Combined oral contraceptives (COCPs)** → Cycle regulation, reduced pain/bleeding.
  - **Oral/Injectable progestins** (e.g., dienogest, depot medroxyprogesterone) → Improve pain and bleeding.
  - **GnRH agonists/antagonists** → Induce hypoestrogenic state, shrink adenomyotic tissue, reduce pain/bleeding. Usually short-term (pre-op or bridging to menopause).
  - **Aromatase inhibitors (letrozole, anastrozole)** → Sometimes used in refractory cases.
- **Tranexamic acid** (during menses) → Reduces heavy bleeding but doesn't treat underlying disease.

---

2. Minimally Invasive Procedures

- **Uterine artery embolization (UAE):**
  - Can reduce adenomyosis volume and symptoms.
  - More effective for focal than diffuse adenomyosis.
  - Fertility impact is uncertain.
- **High-intensity focused ultrasound (HIFU/MRgFUS):**
  - Noninvasive ablation of adenomyotic tissue.
  - Still limited availability, best for focal disease.

---

3. Surgical Options

- **Conservative surgery (adenomyomectomy):**
  - Excision of adenomyotic tissue while preserving the uterus.
  - Technically challenging, risk of recurrence, and uterine rupture in pregnancy.
  - Reserved for severe symptoms in women strongly desiring fertility.
- **Hysterectomy (definitive treatment):**
  - Most effective option for refractory adenomyosis in women who do not desire future fertility.
  - Completely resolves symptoms.

✅ **Summary by patient profile:**

- **Mild symptoms, fertility desired:** NSAIDs, LNG-IUS, or hormonal therapy.
- **Severe symptoms, fertility desired:** Hormonal therapy → Adenomyomectomy (specialized centers) or consider UAE/HIFU.
- **No fertility desire, severe symptoms:** Hysterectomy (definitive).

Would you like me to also make a **comparison table (treatment vs effect on bleeding, pain, fertility, recurrence, invasiveness)** like we did for fibroids? That might be handy for quick counseling in clinic.

Supplementary File 2. ChatGPT's Responses

What are the treatment options for a patient with postpartum hemorrhage?

Postpartum hemorrhage (PPH) is one of the leading obstetric emergencies, and management must be **rapid, stepwise, and multidisciplinary**.

Treatment Options for Postpartum Hemorrhage (PPH)

(defined as blood loss ≥500 mL after vaginal birth or ≥1000 mL after cesarean, or any blood loss causing hemodynamic instability)

---

1. Immediate General Measures

- **Call for help** – obstetric team, anesthesiology, blood bank.
- **Resuscitation**:
  - Two large-bore IV lines
  - IV crystalloids ± blood products (packed RBCs, FFP, platelets; consider MTP = massive transfusion protocol)
  - Oxygen, monitoring (BP, HR, SpO_2, urine output)

---

2. Identify and Treat the Cause

Remember the **"4 T's"**:

- **Tone** (uterine atony – most common)
- **Tissue** (retained placenta or membranes)
- **Trauma** (lacerations, uterine rupture, hematoma)
- **Thrombin** (coagulopathy)

---

3. Uterine Atony Management (Most Common Cause)

Non-surgical measures:

- **Uterine massage** (fundal massage, bimanual compression)
- **Uterotonic drugs (sequentially):**
  - **Oxytocin** (first-line) – 10 IU IM or IV bolus, then infusion
  - **Ergometrine** (contraindicated in hypertension/preeclampsia)

  - **Carboprost (15-methyl $PGF_2\alpha$, Hemabate)** – avoid in asthma
  - **Misoprostol (PGE_1 analogue)** – rectal, sublingual, or oral (useful where other agents unavailable)
- **Tranexamic acid (TXA):**
  - 1 g IV within 3 hours of onset of PPH (WHO recommendation), repeat after 30 min if bleeding continues.
- **Empty bladder** (catheterization improves uterine contraction).

---

4. If Bleeding Persists

- **Mechanical methods:**
  - Intrauterine balloon tamponade (Bakri balloon, Sengstaken tube, condom catheter).
  - Uterine packing (less preferred vs balloon).
- **Surgical interventions (stepwise):**
  - Compression sutures (B-Lynch suture)
  - Bilateral uterine/ovarian artery ligation
  - Internal iliac artery ligation (requires skill)
  - Hysterectomy (definitive, life-saving when all else fails)

---

5. Address Other Causes

- **Retained tissue** → Manual removal of placenta, curettage under anesthesia.
- **Trauma** → Repair of cervical, vaginal, perineal tears; manage uterine rupture.
- **Coagulopathy** → Correct with blood products (FFP, platelets, cryoprecipitate, fibrinogen concentrate).

---

6. Adjuncts

- Recombinant factor VIIa (rare, refractory cases, with caution)
- Cell salvage (intraoperative) if available
- ICU support for ongoing hemodynamic instability

✅ Summary (Stepwise):

1. **Resuscitate + Identify cause (4 T's)**
2. **Uterotonics + TXA + massage**
3. **Balloon tamponade / surgical options if refractory**
4. **Hysterectomy = last resort**

Would you like me to put this into a **visual stepwise algorithm/flow

Supplementary File 2. ChatGPT's Responses

What are the treatment options for a pregnant patient with uterine fibroids?

Good question — **uterine fibroids in pregnancy** can complicate management because both maternal and fetal safety must be considered. Treatment is usually **conservative during pregnancy**, with surgical options generally postponed until after delivery unless there's a life-threatening complication.

Treatment Options for Pregnant Patients with Uterine Fibroids

---

1. Conservative / Supportive Management (mainstay)

- **Observation** → Most women with fibroids in pregnancy are asymptomatic and need only monitoring.
- **Pain management** (for fibroid degeneration, torsion, or pressure symptoms):
    - First-line: **Acetaminophen (paracetamol)**
    - Short courses of **NSAIDs** may be used in the **2nd trimester only** (avoid after 32 weeks due to risk of premature ductus arteriosus closure and oligohydramnios).
    - **Opioids** if pain is severe and unresponsive to other measures.
- **Rest, hydration, supportive care** for pain crises.
- **Monitor pregnancy** → Growth scans, watch for preterm labor, malpresentation, placental abruption, or fetal growth restriction (depending on fibroid size and location).

---

2. Medical Options

- **Uterotonics / GnRH analogues / SPRMs → Contraindicated in pregnancy.**
- **Hormonal therapy** is not used.

---

3. Surgical Options

- **Myomectomy during pregnancy**:
    - Generally **avoided** due to risk of hemorrhage and pregnancy loss.
    - May be considered **only in exceptional cases** (e.g., pedunculated or subserosal fibroid undergoing torsion/necrosis with intractable pain, or obstructing the uterus causing complications).

- **Myomectomy at cesarean section**:
    - Traditionally avoided due to risk of massive hemorrhage.
    - Increasingly reported as **safe in selected cases** (especially pedunculated subserosal fibroids).
    - Decision is individualized depending on fibroid size, location, and surgical expertise.
- **Hysterectomy during pregnancy** → Reserved for uncontrolled life-threatening hemorrhage (very rare, catastrophic scenarios).

---

4. Postpartum Management

- **Reassess fibroid size** (many shrink postpartum).
- **Definitive treatment** (myomectomy, UAE, hysterectomy) can be planned after completion of pregnancy and recovery.

✅ **Summary:**

- **During pregnancy** → conservative management is standard (analgesia, monitoring, supportive care).
- **Surgery** only for rare complications (torsion, necrosis, obstruction).
- **Definitive treatment** is postponed to postpartum or combined with cesarean only in selected, safe scenarios.

Would you like me to also prepare a **table of pregnancy complications associated with fibroids (e.g., miscarriage, malpresentation, preterm labor, PPH, C-section rates) and how we manage them**? That could give you a quick reference for exams or ward work.

Supplementary File 2. ChatGPT's Responses

What are the treatment options for a patient with uterine fibroids and fertility wish?

That's a key clinical scenario — **uterine fibroids with fertility desire**. Management needs to balance **symptom relief, fertility preservation, and minimizing recurrence**.

Treatment Options for Uterine Fibroids in Patients Desiring Fertility

---

1. Expectant Management

- If fibroids are **small, asymptomatic, not distorting the endometrial cavity**, and the woman is otherwise fertile → **watchful waiting** with regular monitoring (ultrasound, symptoms).

---

2. Medical Therapy (bridging, not definitive for fertility)

- **Not a long-term solution for fertility**, but may be used preoperatively to optimize the patient (e.g., correct anemia, shrink fibroids).
- **GnRH agonists/antagonists** → temporary shrinkage, improve anemia; helpful before myomectomy but symptoms recur after discontinuation.
- **Ulipristal acetate (SPRM)** → reduces bleeding and size, but restricted in many countries due to hepatotoxicity concerns.
- **Tranexamic acid / NSAIDs / hormonal contraceptives** → help symptoms but **do not improve fertility**.
  📌 **Medical therapy is generally used as a bridge to surgery, not as stand-alone fertility treatment.**

---

3. Surgical Options (mainstay for fertility preservation)

- **Myomectomy (uterus-preserving surgery)**
  - **Hysteroscopic myomectomy** → for submucosal fibroids (best evidence for improving fertility).
  - **Laparoscopic myomectomy** → for intramural or subserosal fibroids of moderate size.

- **Open myomectomy** → for very large or multiple fibroids.
- Reduces miscarriage risk and improves pregnancy rates, especially if cavity is distorted.
- Risk: adhesions, recurrence (up to 60% at 5 years).

---

4. Minimally Invasive / Interventional Radiology

- **Uterine Artery Embolization (UAE):**
  - Usually **not recommended in women desiring fertility**, as it may impair ovarian reserve and increase pregnancy complications (miscarriage, preterm birth, abnormal placentation).
- **MRI-guided Focused Ultrasound (MRgFUS):**
  - Limited data on fertility outcomes; not first-line if pregnancy is a goal.

---

5. Assisted Reproductive Techniques (ART)

- For women with fibroids plus other infertility factors → **IVF/ICSI** may be considered.
- Submucosal or large intramural fibroids **should be removed first** to optimize implantation and reduce miscarriage.

---

6. Definitive Surgery (Not Suitable if Fertility Desired)

- **Hysterectomy** → contraindicated in fertility wish.

✅ **Summary by Fibroid Type (with fertility wish):**

- **Submucosal fibroids (distorting cavity)** → *Hysteroscopic myomectomy* (clear benefit for fertility).
- **Intramural fibroids >4 cm, distorting cavity** → *Laparoscopic/open myomectomy*.
- **Subserosal fibroids** → *Usually don't affect fertility*, remove only if large/symptomatic.
- **UAE / MRgFUS** → *Avoid if fertility desired*.

Supplementary File 2. ChatGPT's Responses

Would you like me to draw up a **flowchart: "Fibroids + fertility wish → management decision-making by fibroid type and symptoms"** for you? That would be handy for both clinical reasoning and exams.

Supplementary File 2. ChatGPT's Responses

What are the treatment options for a patient with uterine fibroids and active pelvic infection?

That's a very tricky situation — **uterine fibroids + active pelvic infection**. The infection takes priority, because **any surgical or interventional fibroid treatment in the presence of infection carries a very high risk of sepsis and morbidity**.

Treatment Options for Uterine Fibroids with Active Pelvic Infection

---

1. Stabilize & Treat Infection First

- **Broad-spectrum IV antibiotics** (covering aerobes/anaerobes, e.g. cephalosporin + doxycycline + metronidazole, or clindamycin + gentamicin).
- **Hospitalization** if severe systemic illness, abscess, or sepsis.
- **Drainage** (surgical or image-guided) if pelvic abscess present.
- **Supportive care**: fluids, analgesia, correction of anemia if present.

---

2. Fibroid-Specific Management (only *after infection resolves*)

- **Medical management** (safe while infection is active, but limited effect on fibroid bulk):
    - NSAIDs for pain (unless contraindicated).
    - Tranexamic acid for bleeding (if menorrhagia present).
    - Short-term hormonal therapy (e.g. GnRH agonists) can be considered later, once infection is cleared.
- **Surgical management** (myomectomy, hysterectomy):
    - **Contraindicated during active infection** (risk of sepsis, wound breakdown).
    - Can be performed **electively** once infection has been fully treated and the patient stabilized.
    - In rare cases of **fibroid infection/pyomyoma → urgent surgery (myomectomy or hysterectomy)** may be required in addition to antibiotics.
- **Uterine Artery Embolization (UAE):**
    - **Contraindicated in active pelvic infection**, since ischemic tissue necrosis could worsen sepsis.
    - Can be reconsidered later if uterus preservation is desired.

---

3. Special Scenario: Pyomyoma (Infected Fibroid)

- Rare but serious (life-threatening sepsis risk).
- Presents with **fever, leukocytosis, abdominal pain, sepsis signs** in a patient with fibroids.
- **Management**: Broad-spectrum IV antibiotics **+ urgent surgery** (myomectomy or hysterectomy, depending on fertility wish and surgical feasibility).

✅ **Summary:**

- **First priority = eradicate infection** (antibiotics ± drainage).
- **Do not attempt elective fibroid surgery or UAE until infection is resolved.**
- If infection involves the fibroid itself (pyomyoma) → antibiotics + urgent surgical removal.

Would you like me to also create a **quick "Do vs Don't" table** for fibroid management in the setting of active infection, so it's easy to recall in exams or ward rounds?

Supplementary File 2. ChatGPT’s Responses

What are the treatment options for a patient with adenomyosis and a suspicious adnexal mass?

This is a complex scenario because we are dealing with **two gynecologic problems at once**:

- **Adenomyosis** → usually a benign, chronic condition causing heavy bleeding and pelvic pain.
- **Suspicious adnexal mass** → possible ovarian malignancy (must be prioritized).

Treatment Options: Adenomyosis + Suspicious Adnexal Mass

---

1. Prioritization of Management

- **The adnexal mass takes priority** since malignancy must be ruled out/treated urgently.
- Management of adenomyosis is generally deferred until after the adnexal pathology has been clarified or treated.

---

2. Work-up Before Treatment

- Full **pelvic imaging** (transvaginal US ± MRI).
- **Tumor markers** (CA-125, HE4, others depending on risk profile).
- **Referral to gynecologic oncology** if the adnexal mass is highly suspicious.

---

3. Definitive Surgical Management

- **If fertility not desired** (common in women with adenomyosis, often >40):
  - **Total hysterectomy with bilateral salpingo-oophorectomy (BSO)** is often chosen → treats adenomyosis and removes the adnexal mass in one operation.
  - Oncologic staging procedure if malignancy is confirmed or strongly suspected.
- **If fertility is desired / premenopausal woman:**
  - Management must be individualized.

    - **Suspicious adnexal mass**: unilateral salpingo-oophorectomy or ovarian cystectomy (depending on oncologic safety and intraoperative findings).
    - **Adenomyosis**: conservative measures (medical therapy such as LNG-IUS, progestins, or GnRH analogues) until after fertility is achieved.
    - Adenomyomectomy can be considered later if symptoms are severe and fertility must be preserved, but technically challenging.

---

4. Medical Management of Adenomyosis (only if adnexal mass ruled out benign)

- **NSAIDs** for pain.
- **Hormonal options**:
    - Levonorgestrel IUS (first-line for bleeding and pain).
    - Oral progestins or dienogest.
    - Combined oral contraceptives.
    - GnRH agonists/antagonists (short-term).
- **UAE or HIFU**: options for symptom control in women not suitable for hysterectomy but fertility not a priority.

✅ **Summary:**

- **Suspicious adnexal mass takes precedence** → evaluate/operate with oncologic principles.
- **If fertility complete:** hysterectomy ± BSO is often definitive and addresses both adenomyosis and adnexal mass.
- **If fertility desired:** prioritize safe management of the adnexal mass; manage adenomyosis medically or conservatively until after fertility.

Would you like me to make a **decision tree (fertility desire vs malignancy risk)** for this scenario? That would show clearly which path to take depending on the patient profile.

Supplementary File 2. ChatGPT's Responses

What are the treatment options for a patient with pedunculated uterine fibroids?

Pedunculated fibroids are a special subgroup of **uterine leiomyomas** because they are attached to the uterus by a stalk. Their management depends on **location (subserosal vs submucosal), symptoms, and fertility wishes**.

Treatment Options for Pedunculated Uterine Fibroids

---

1. Expectant (Conservative) Management

- **Asymptomatic, small fibroids** → observation with periodic ultrasound.
- Indicated if no pressure, bleeding, or fertility issues.

---

2. Medical Therapy (Symptom Control, Not Curative)

- **For bleeding/pain:**
  - NSAIDs (for dysmenorrhea/pain).
  - Tranexamic acid (for heavy menstrual bleeding).
  - Hormonal therapies: LNG-IUS, combined OCPs, progestins.
- **Fibroid shrinkage (temporary):**
  - GnRH agonists/antagonists → used preoperatively to reduce size/vascularity.
- 📌 Medical therapy **does not eliminate pedunculated fibroids** and is usually a bridge to surgery.

---

3. Surgical Options (Definitive Management)

- **Myomectomy (preferred for fertility preservation):**
  - **Subserosal pedunculated fibroid:**
    - Can often be removed laparoscopically (straightforward because of stalk).
    - Risk: torsion (causing acute pain/necrosis) → indication for removal.
  - **Submucosal pedunculated fibroid:**
    - Best removed hysteroscopically (minimally invasive, preserves uterus).

- **Hysterectomy:**
  - Definitive option if multiple fibroids, large symptomatic fibroids, or no fertility desire.

---

4. Minimally Invasive Interventions

- **Uterine Artery Embolization (UAE):**
  - **Generally avoided** for pedunculated subserosal fibroids because of risk of stalk necrosis, detachment, and peritonitis.
- **MRI-guided Focused Ultrasound (MRgFUS):**
  - Technically possible but less effective for pedunculated fibroids, especially if subserosal.

---

5. Emergency Situations

- **Torsion of a pedunculated fibroid (especially subserosal):**
  - Presents with acute abdomen, severe pain, possible peritonitis.
  - Requires **urgent surgical removal (myomectomy or hysterectomy)**.

✅ **Summary by Fibroid Type:**

- **Subserosal pedunculated fibroid:** laparoscopic myomectomy (avoid UAE).
- **Submucosal pedunculated fibroid:** hysteroscopic myomectomy.
- **Asymptomatic:** observation.
- **No fertility wish + multiple symptoms:** hysterectomy.
- **Complication (torsion, necrosis):** urgent surgery.

Would you like me to make you a **table comparing subserosal vs submucosal pedunculated fibroids (presentation, complications, and treatment approach)** for quick reference?
