# Supplementary material for: The Role of Large Language Models in the Promotion of Minimally Invasive Interventional Radiologic Methods in Gynecology and Obstetrics
Source: J Clin Med. 2026 Apr 23;15(9):3234. doi: 10.3390/jcm15093234 (PMC13163413; doi:10.3390/jcm15093234)
Supplement: Supplementary file 1 [file jcm-15-03234-s001.zip › S3-OpenEvidence UAE.pdf]

Supplementary File 3. OpenEvidence's Responses

What are the treatment options for a patient suffering from menorrhagia due to uterine fibroids?

According to an invited review in the New England Journal of Medicine, **the treatment options for menorrhagia due to uterine fibroids include medical therapies, minimally invasive procedures, and surgical interventions**. First-line medical management typically involves **contraceptive hormones** (oral, patch, implant, or levonorgestrel intrauterine device), which are recommended to control heavy menstrual bleeding, although the supporting evidence is of low quality and efficacy may be limited in patients with large or submucosal fibroids. **Nonsteroidal anti-inflammatory drugs (NSAIDs)** and **tranexamic acid** are also used, but their efficacy is better established for idiopathic heavy menstrual bleeding rather than fibroid-related menorrhagia.

A major advance in medical therapy is the use of **oral gonadotropin-releasing hormone (GnRH) antagonist combinations** (elagolix, relugolix, or linzagolix, each with estradiol and progestin), which rapidly suppress ovarian steroidogenesis and can induce amenorrhea in approximately half of patients, with a significant reduction in uterine volume and heavy bleeding. These agents are approved for up to 24 months of use in the United States (except linzagolix) and are associated with fewer hypogonadal symptoms compared to GnRH agonists.

GnRH agonists (e.g., depot leuprolide) are approved for short-term preoperative use, leading to amenorrhea in most patients and substantial reduction in uterine size, but are limited by side effects such as bone loss and hot flushes. Surgical options, including hysterectomy, remain common but are associated with long-term sequelae; therefore, alternatives such as uterine-artery embolization, focused ultrasound ablation, and radiofrequency ablation should be considered, especially for patients seeking uterine preservation.

The rationale for these recommendations is to balance efficacy in reducing heavy menstrual bleeding and fibroid size with the risk profile and patient preferences, especially regarding fertility preservation and avoidance of major surgery.

Building on the prior discussion of medical and surgical options, recent consensus and guideline updates further clarify the approach to **menorrhagia due to uterine fibroids**. The American College of Radiology recommends that initial therapy should be tailored to the patient's reproductive goals, fibroid characteristics, and symptom severity. For patients desiring uterine preservation or future fertility, **first-line medical management** includes **estrogen-progestin oral contraceptives** and **progestin-containing intrauterine devices (IUDs)**, both of which can reduce bleeding symptoms. **Tranexamic acid** is a nonhormonal alternative, taken only during menses, and is effective in reducing menstrual blood loss, but should not be used concurrently with oral contraceptives due to thrombotic risk.

For patients with more severe symptoms or those preparing for surgery, **GnRH agonists** (e.g., leuprolide acetate) and **oral GnRH antagonists** (elagolix, relugolix, linzagolix) are effective at reducing both bleeding and fibroid volume. These agents are typically used short-term due to hypoestrogenic side effects, which can be mitigated by "add-back" therapy with low-dose estrogen and progestin—a regimen now FDA-approved for up to 24 months for fibroid-related heavy menstrual bleeding.The use of **selective progesterone receptor modulators (SPRMs)**, such as ulipristal acetate, has shown efficacy in reducing bleeding and fibroid size, but its use is currently restricted due to concerns about hepatotoxicity.

Supplementary File 3. OpenEvidence's Responses

When medical therapy fails or is contraindicated, **uterine-sparing procedures** such as **hysteroscopic myomectomy** (for submucosal fibroids), **uterine artery embolization**, and **magnetic resonance-guided focused ultrasound** are recommended, with the choice guided by fibroid location, size, and patient preference. For women who do not desire future fertility or have refractory symptoms, **hysterectomy** remains definitive. Management of iron deficiency and anemia is essential throughout, with iron supplementation recommended as needed.

In summary, the treatment algorithm is individualized, with the American College of Radiology and recent systematic reviews supporting a stepwise approach from medical therapy to minimally invasive and surgical interventions, always considering patient goals and safety.

Supplementary File 3. OpenEvidence's Responses

What are the treatment options for a patient with uterine fibroids and pelvic pain?

For a patient with **uterine fibroids and pelvic pain**, treatment options should be individualized based on symptom severity, fibroid characteristics, reproductive goals, and patient preference. First-line medical therapies include **nonsteroidal anti-inflammatory drugs (NSAIDs)**, which provide moderate pain relief and modest reduction in menstrual bleeding, and **hormonal contraceptives** (oral, patch, or levonorgestrel-releasing intrauterine device), which can reduce bleeding and may help with pain, though evidence for pain reduction is less robust.

For patients with more severe symptoms, **oral GnRH antagonist combinations** (e.g., elagolix or relugolix with estradiol and norethindrone acetate) are FDA-approved for up to 24 months and have demonstrated significant reductions in pain (10–40%), heavy menstrual bleeding, and modest reduction in uterine volume, with a favorable side-effect profile compared to GnRH agonists. **GnRH agonists** (e.g., depot leuprolide) are approved for short-term preoperative use and can improve pain and reduce uterine size, but are limited by hypogonadal side effects and bone loss.

Selective progesterone-receptor modulators (e.g., ulipristal acetate) are available in some countries and can improve pain and reduce fibroid volume, but are not approved in the United States due to concerns about rare hepatic toxicity.

Surgical and interventional options for persistent pain or bulk symptoms include **myomectomy**(hysteroscopic, laparoscopic, or abdominal), **uterine-artery embolization**, **MRI-guided focused ultrasound ablation**, and **radiofrequency ablation**. These procedures are chosen based on fibroid size, number, and location, and can provide substantial symptom relief while preserving the uterus.

Shared decision-making is essential, considering the patient's reproductive goals and preferences, as well as the risk-benefit profile of each therapy.

Supplementary File 3. OpenEvidence's Responses

What are the treatment options for a patient with uterine fibroids and urinary/stool incontinence?

For a patient with **uterine fibroids and urinary or fecal incontinence**, management should be individualized and address both fibroid-related bulk symptoms and the incontinence. Fibroids can cause urinary frequency, urgency, and, less commonly, incontinence or bowel dysfunction due to mass effect. The American College of Obstetricians and Gynecologists and the Society for Obstetricians and Gynecologists of Canada recommend that initial management of symptomatic fibroids should be medical, with interventional options considered for persistent or severe symptoms.

First-line treatment for symptomatic fibroids includes medical therapy such as oral GnRH antagonist combinations (e.g., relugolix with estradiol and norethindrone acetate), which can reduce bulk-related symptoms and bleeding, with modest reduction in uterine volume and a favorable side-effect profile. Hormonal contraceptives, tranexamic acid, and nonsteroidal anti-inflammatory drugs may also be used for bleeding, but are less effective for bulk symptoms.

If medical therapy does not adequately relieve bulk symptoms causing incontinence, **uterine-sparing interventional procedures** are indicated. Uterine artery embolization, myomectomy (hysteroscopic, laparoscopic, or abdominal), MRI-guided focused ultrasound, and radiofrequency ablation are all effective in reducing fibroid size and alleviating pressure effects, including urinary and bowel symptoms. The choice depends on fibroid size, location, reproductive goals, and patient preference.

For **urinary or fecal incontinence**, standard pelvic floor therapy, behavioral modifications, and, if indicated, urologic or colorectal evaluation should be pursued in parallel, as fibroid removal may not fully resolve incontinence if other etiologies are present.

In summary, **medical therapy is first-line for symptomatic fibroids**, with minimally invasive procedures reserved for persistent bulk symptoms affecting urinary or bowel function. Management should be multidisciplinary and tailored to symptom severity, fibroid characteristics, and patient goals.

What are the treatment options for a patient with adenomyosis?

Treatment options for a patient with adenomyosis include both medical and surgical approaches, with the choice guided by symptom severity, reproductive goals, and patient preference. First-line therapy is typically **hormonal management**, with the **levonorgestrel-releasing intrauterine system (LNG-IUS)** considered the most effective for controlling heavy menstrual bleeding and dysmenorrhea, and for reducing uterine volume. Progestins such as **dienogest** are also effective, particularly for pain control, and have demonstrated superior reduction in dysmenorrhea compared to other medical treatments. Combined oral contraceptives (COCs) may be used, but are generally less effective than progestins or LNG-IUS for symptom control.
Gonadotropin-releasing hormone (GnRH) agonists are effective for symptom relief and uterine volume reduction, but their use is limited by hypoestrogenic side effects and is generally reserved for short-term or second-line therapy. Emerging options include **GnRH antagonists** and **aromatase inhibitors**, which may be considered in refractory cases, though data on long-term safety and efficacy are limited.
For patients who do not respond to medical therapy or who desire definitive treatment, **hysterectomy**remains the only curative option. Conservative surgical approaches (e.g., adenomyomectomy) and minimally invasive procedures such as **high-intensity focused ultrasound (HIFU)** or **uterine artery embolization (UAE)** may be considered for uterine preservation, but should be performed in specialized centers due to complexity and potential obstetric risks.
Non-hormonal options such as **NSAIDs** and **tranexamic acid** may provide symptomatic relief for mild cases, but do not address the underlying pathology.There are currently no disease-specific guidelines from major societies, and management should be individualized based on the available evidence.

What are the treatment options for a patient with postpartum hemorrhage?

Treatment options for a patient with postpartum hemorrhage should be initiated promptly and follow a stepwise approach, prioritizing rapid identification and management of the underlying cause.The most common etiology is uterine atony.
First-line therapy is **uterine massage** combined with **oxytocin** (IV: 10–40 IU in 500–1000 mL lactated Ringer's; IM: 5–10 IU, up to 4 doses), which stimulates uterine contractions and is the mainstay of initial management. If oxytocin is insufficient, **second-line uterotonics** include **methylergonovine maleate** (IM: 0.2 mg every 2–4 hr, max 5 doses; contraindicated in hypertension/cardiovascular disease) and **carboprost tromethamine** (IM: 250 µg every 15–90 min, max 8 doses; contraindicated in asthma). Misoprostol (600–1000 µg, sublingual preferred) may be considered, but its efficacy is disputed.
Adjunctive therapy includes **tranexamic acid** (IV: 1 g over 10 min, repeatable after 30 min if bleeding persists), which should be administered early and has demonstrated mortality benefit. Recombinant factor VIIa may be considered in refractory cases.
If pharmacologic measures fail, **mechanical interventions** such as **intrauterine balloon tamponade**(e.g., Bakri balloon) or **uterine compression sutures** (e.g., B-Lynch) are indicated. Surgical options include **uterine artery ligation**, **internal iliac artery ligation**, and ultimately **hysterectomy** if conservative measures are unsuccessful.
Supportive care involves volume resuscitation with crystalloids, blood products (packed red cells, fresh-frozen plasma, platelets, cryoprecipitate as indicated by laboratory values), and correction of coagulopathy. Early activation of massive transfusion protocols and hemostatic resuscitation may be required in severe cases.
Management should always be multidisciplinary and escalate rapidly if initial measures fail.

What are the treatment options for a pregnant patient with uterine fibroids?

Supplementary File 3. OpenEvidence's Responses

For a **pregnant patient with uterine fibroids**, the American College of Radiology states that most medical management options commonly used for fibroid symptoms—such as estrogen-progestin oral contraceptives, progestin-containing intrauterine devices, tranexamic acid, gonadotropin-releasing hormone (GnRH) agonists/antagonists, and progesterone receptor modulators—are contraindicated or not recommended during pregnancy due to potential risks to the fetus and lack of safety data in pregnancy.

The primary approach during pregnancy is **conservative management**, focusing on symptomatic relief and close monitoring. This includes analgesia with acetaminophen for pain, as NSAIDs are generally avoided in pregnancy, especially in the third trimester due to risks of premature closure of the ductus arteriosus. Surgical intervention (e.g., myomectomy) is reserved for rare, severe cases such as intractable pain, torsion of a pedunculated fibroid, or complications like obstruction or degeneration, and is generally avoided due to increased risk of pregnancy loss and preterm labor.

The rationale for this conservative approach is to minimize maternal and fetal risk, as most fibroids do not require intervention during pregnancy and many patients remain asymptomatic.The American College of Radiology emphasizes that medical therapies for fibroids are intended for non-pregnant patients and those desiring fertility preservation outside of pregnancy.

Building on the conservative management approach outlined above, the medical literature further emphasizes that **active intervention for uterine fibroids during pregnancy is rarely indicated**. Most fibroids remain asymptomatic or cause only mild symptoms, and the majority of pregnant patients do not require any specific treatment beyond observation and supportive care. When symptoms do arise, such as pain from fibroid degeneration, the recommended management is supportive, including hydration, rest, and acetaminophen for analgesia. NSAIDs may be considered in the second trimester for short courses, but are generally avoided in the third trimester due to fetal risks.

Surgical intervention, specifically **myomectomy during pregnancy**, is reserved for exceptional circumstances such as torsion of a pedunculated subserosal fibroid, severe intractable pain unresponsive to medical therapy, or life-threatening complications like bowel or urinary obstruction.Even in these cases, surgery carries significant risks, including miscarriage, preterm labor, and hemorrhage, and is generally deferred unless absolutely necessary. Uterine artery embolization, focused ultrasound, and other interventional radiologic procedures are contraindicated in pregnancy due to risks to both mother and fetus.

There is no role for hormonal therapies, selective progesterone receptor modulators, or GnRH analogs during pregnancy, as these agents are either contraindicated or lack safety data in pregnant populations. The literature consistently supports a **watchful waiting approach**, with individualized care based on symptom severity and maternal-fetal status. Iron supplementation may be indicated if anemia develops secondary to fibroid-related bleeding, though this is uncommon in pregnancy.

In summary, the consensus across clinical trials and reviews is that **conservative management and symptomatic relief** are the mainstays of treatment for uterine fibroids in pregnancy, with surgical intervention reserved for rare, severe complications.

What are the treatment options for a patient with uterine fibroids and fertility wish?

Supplementary File 3. OpenEvidence's Responses

For a patient with uterine fibroids who wishes to preserve fertility, the American College of Radiology recommends several treatment options, with selection based on symptom severity, fibroid characteristics, and reproductive goals. **First-line medical management** includes estrogen-progestin oral contraceptive pills and progestin-containing intrauterine devices, which primarily reduce bleeding symptoms. Nonhormonal agents such as tranexamic acid may also be considered for bleeding control. These options do not reduce fibroid size but are uterus-sparing and do not impair future fertility, though contraceptive effects are present during use.

Second-line medical therapy involves gonadotropin-releasing hormone (GnRH) agonists (e.g., leuprolide acetate) and oral GnRH antagonists (e.g., elagolix, linzagolix, relugolix), which can reduce both bleeding and fibroid volume. These agents are typically used short-term, often preoperatively, due to hypoestrogenic side effects and rapid symptom recurrence after discontinuation. Combination regimens with low-dose estrogen and progestin can mitigate adverse effects and are FDA-approved for heavy menstrual bleeding related to fibroids.

Surgical options include hysteroscopic myomectomy for submucosal fibroids and laparoscopic or open myomectomy for larger or intramural fibroids. These procedures are uterus-sparing and considered standard for women desiring future fertility. The American College of Radiology also notes that MR-guided focused ultrasound ablation and uterine artery embolization may be appropriate in select cases, though the impact on fertility should be carefully considered and discussed.

Progesterone receptor modulators (e.g., ulipristal acetate) are emerging options that can reduce both bleeding and bulk symptoms, with intermittent dosing allowing for menstruation during breaks, but their use in the U.S. is limited and should be individualized.

Building on the above, recent clinical trials and systematic reviews have further clarified the efficacy and reproductive safety of minimally invasive, uterus-sparing interventions for **patients with uterine fibroids who wish to preserve fertility**. Myomectomy remains the most established surgical approach, with hysteroscopic myomectomy preferred for submucosal fibroids and laparoscopic or open myomectomy for intramural or subserosal lesions. These techniques are associated with improved pregnancy rates and are considered standard for women with fibroid-related infertility, as supported by contemporary reviews and meta-analyses.

Minimally invasive radiologic procedures, including **uterine artery embolization (UAE)**, **high-intensity focused ultrasound (HIFU)**, and **transcervical radiofrequency ablation (TFA)**, have demonstrated comparable live birth rates to surgical myomectomy in select populations, though the miscarriage rate is higher after UAE and placental abnormalities are more frequent after HIFU. The available evidence suggests that these modalities may be considered for women who desire uterine preservation, but counseling regarding potential reproductive risks is essential, and long-term data remain limited, especially for TFA. The choice among these options should be individualized based on fibroid characteristics, patient age, and reproductive goals.

Medical therapies, including **GnRH antagonists** (elagolix, relugolix, linzagolix), have shown rapid reduction in fibroid volume and improvement in heavy menstrual bleeding, with a reduction in fibroid size of up to 50% over 24 weeks. However, their use is typically short-term and primarily as a bridge to surgery or for symptom control, given the risk of hypoestrogenic side effects and the lack of long-term safety data regarding fertility outcomes. Selective progesterone receptor modulators (SPRMs), such as ulipristal acetate, are effective for symptom control and volume reduction, but

regulatory restrictions and concerns about endometrial safety limit their use in some regions.
In summary, **myomectomy** is the preferred intervention for women with fibroid-related infertility, while minimally invasive procedures and medical therapies may be considered in select cases, with shared decision-making and individualized risk assessment.

What are the treatment options for a patient with uterine fibroids and active pelvic infection?

The first-line treatment for a patient with uterine fibroids and an active pelvic infection is to address the pelvic infection before initiating any fibroid-specific interventions. According to the United States Centers for Disease Control and Prevention (CDC) guidelines, empiric, broad-spectrum antimicrobial therapy should be started promptly for pelvic inflammatory disease (PID), which is the most common cause of active pelvic infection in this context. Recommended regimens include **ceftriaxone 500 mg intramuscularly in a single dose, plus doxycycline 100 mg orally twice daily for 14 days, with metronidazole 500 mg orally twice daily for 14 days**. Alternative regimens include cefoxitin 2 g IM plus probenecid 1 g orally in a single dose, with the same doxycycline and metronidazole schedule.

Surgical or interventional treatments for uterine fibroids—such as myomectomy, hysterectomy, uterine artery embolization, or ablation—are **contraindicated in the presence of active pelvic infection** due to the risk of exacerbating infection and causing severe complications. Specifically, uterine artery embolization is absolutely contraindicated in patients with active infection. Medical therapies for fibroid symptoms (e.g., hormonal agents, NSAIDs, tranexamic acid) may be considered for symptom control, but intrauterine devices (e.g., levonorgestrel IUD) should be avoided until infection is resolved.

Once the infection is adequately treated and resolved, fibroid management can be individualized based on symptoms, reproductive goals, and fibroid characteristics. In summary, **treat the pelvic infection first with CDC-recommended antibiotics; defer any fibroid-specific surgical or interventional procedures until infection is cleared**.

What are the treatment options for a patient with adenomyosis and a suspicious adnexal mass?

Supplementary File 3. OpenEvidence’s Responses

For a patient with **adenomyosis and a suspicious adnexal mass**, management must address both conditions, with priority given to the evaluation and management of the adnexal mass due to the potential for malignancy.

•*1. Suspicious Adnexal Mass:**

The primary concern is to rule out ovarian or fallopian tube malignancy. The medical literature emphasizes that all patients with high-risk adnexal lesions should be referred to a gynecologic oncologist for evaluation and treatment. Management typically involves surgical exploration, with the extent of surgery (cystectomy, oophorectomy, or staging procedure) determined by intraoperative findings, patient age, fertility desires, and malignancy risk assessment. Preoperative workup includes transvaginal ultrasound, serum CA-125, and possibly MRI, but imaging cannot reliably exclude malignancy. Observation is only appropriate for simple, unilocular, likely benign lesions; complex or suspicious masses require surgical management.

•*2. Adenomyosis:**

Definitive treatment for adenomyosis is hysterectomy, which may be appropriate if the patient has completed childbearing and is undergoing surgery for the adnexal mass. For those desiring uterine preservation, first-line therapy is hormonal management, most commonly with a levonorgestrel-releasing intrauterine system (LNG-IUS), oral progestins (e.g., dienogest), or gonadotropin-releasing hormone (GnRH) analogs. These options are effective for symptom control but are not disease-modifying and are generally considered when surgery is not indicated or as adjuncts.

Summary:

Surgical management is indicated for a suspicious adnexal mass, with the approach tailored to malignancy risk and patient factors. **Medical or surgical management of adenomyosis** should be individualized, with hysterectomy considered if surgery is already planned and fertility is not desired.Otherwise, hormonal therapies are first-line for symptom control.

What are the treatment options for a patient with pedunculated uterine fibroids?

Supplementary File 3. OpenEvidence's Responses

For a patient with **pedunculated uterine fibroids**, the American College of Radiology recommends **hysteroscopic myomectomy** or **medical management** as appropriate initial treatment options, particularly for reproductive-age patients who are symptomatic with heavy uterine bleeding.

Hysteroscopic myomectomy is considered the procedure of choice for pedunculated submucosal fibroids less than 5 cm, offering shorter hospitalization and faster recovery compared to laparoscopic or open myomectomy, with equivalent improvement in symptoms and quality of life at 2–3 months post-procedure.

Medical management should generally be trialed prior to invasive therapy. First-line agents include NSAIDs and combined estrogen-progestin oral contraceptives to reduce bleeding.Tranexamic acid is a nonhormonal alternative for bleeding control. Second-line options include GnRH agonists (e.g., leuprolide acetate) and oral GnRH antagonists (e.g., elagolix, linzagolix, relugolix), which can reduce both bleeding and fibroid volume, often used short-term preoperatively. These agents may cause hypoestrogenic side effects, which can be mitigated by add-back therapy with low-dose estrogen and progestin. Progesterone receptor modulators (e.g., ulipristal acetate) have demonstrated efficacy but are not approved in the US due to hepatotoxicity concerns.

The rationale for these recommendations is to balance symptom control, minimize invasiveness, and preserve fertility when desired. The American College of Radiology emphasizes that medical management should precede surgical intervention unless rapid symptom relief or definitive treatment is required.

In addition to the initial management strategies, the choice of treatment for **pedunculated uterine fibroids** is further influenced by fibroid location (submucosal vs. subserosal), size, and patient-specific factors such as reproductive goals and comorbidities. For pedunculated submucosal fibroids (FIGO type 0), **hysteroscopic myomectomy** remains the preferred approach due to its efficacy, safety profile, and rapid recovery, as supported by randomized trials and expert consensus. For pedunculated subserosal fibroids (FIGO type 7), **laparoscopic myomectomy** is typically favored, as these lesions are accessible and can be excised with minimal uterine trauma, preserving fertility and reducing bulk symptoms.

Minimally invasive alternatives such as **uterine artery embolization (UAE)**, **radiofrequency ablation (RFA)**, and **high-intensity focused ultrasound (HIFU)** are effective for symptom control and fibroid volume reduction, but are less commonly used for pedunculated fibroids due to the risk of post-procedural expulsion and infection, particularly with UAE. UAE is generally reserved for women who decline surgery or are poor surgical candidates, with the caveat that pedunculated subserosal fibroids may be at higher risk for complications following embolization.

Medical therapy—including NSAIDs, tranexamic acid, combined oral contraceptives, and short-term GnRH agonists or antagonists—remains adjunctive, primarily for symptom control or preoperative optimization, but does not address the underlying fibroid bulk. Selective progesterone receptor modulators (SPRMs) such as ulipristal acetate are effective in reducing symptoms and fibroid size, but their use is limited by regulatory restrictions in the United States.

Definitive management with **hysterectomy** is reserved for women who have completed childbearing or in whom conservative measures have failed, and is associated with the lowest recurrence risk.Shared decision-making is essential, integrating patient preferences, symptom severity, and reproductive plans.
